# Localization of Neuropeptide Gene Expression in Larvae of an Echinoderm, the Starfish *Asterias rubens*

**DOI:** 10.3389/fnins.2016.00553

**Published:** 2016-12-01

**Authors:** Tatiana D. Mayorova, Shi Tian, Weigang Cai, Dean C. Semmens, Esther A. Odekunle, Meet Zandawala, Yusef Badi, Matthew L. Rowe, Michaela Egertová, Maurice R. Elphick

**Affiliations:** ^1^Department of Organismal Biology, School of Biological and Chemical Sciences, Queen Mary University of LondonLondon, UK; ^2^Laboratory of Developmental Neurobiology, Koltzov Institute of Developmental Biology of Russian Academy of SciencesMoscow, Russia

**Keywords:** neuropeptide precursor, mRNA *in situ* hybridization, larval nervous system, bipinnaria, brachiolaria, attachment complex, Echinodermata

## Abstract

Neuropeptides are an ancient class of neuronal signaling molecules that regulate a variety of physiological and behavioral processes in animals. The life cycle of many animals includes a larval stage(s) that precedes metamorphic transition to a reproductively active adult stage but, with the exception of *Drosophila melanogaster* and other insects, research on neuropeptide signaling has hitherto largely focused on adult animals. However, recent advances in genome/transcriptome sequencing have facilitated investigation of neuropeptide expression/function in the larvae of protostomian (e.g., the annelid *Platynereis dumerilii*) and deuterostomian (e.g., the urochordate *Ciona intestinalis*) invertebrates. Accordingly, here we report the first multi-gene investigation of larval neuropeptide precursor expression in a species belonging to the phylum Echinodermata—the starfish *Asterias rubens*. Whole-mount mRNA *in situ* hybridization was used to visualize in bipinnaria and brachiolaria stage larvae the expression of eight neuropeptide precursors: L-type SALMFamide (S1), F-type SALMFamide (S2), vasopressin/oxytocin-type, NGFFYamide, thyrotropin-releasing hormone-type, gonadotropin-releasing hormone-type, calcitonin-type and corticotropin-releasing hormone-type. Expression of only three of the precursors (S1, S2, NGFFYamide) was observed in bipinnaria larvae but by the brachiolaria stage expression of all eight precursors was detected. An evolutionarily conserved feature of larval nervous systems is the apical organ and in starfish larvae this comprises the bilaterally symmetrical lateral ganglia, but only the S1 and S2 precursors were found to be expressed in these ganglia. A prominent feature of brachiolaria larvae is the attachment complex, comprising the brachia and adhesive disk, which mediates larval attachment to a substratum prior to metamorphosis. Interestingly, all of the neuropeptide precursors examined here are expressed in the attachment complex, with distinctive patterns of expression suggesting potential roles for neuropeptides in the attachment process. Lastly, expression of several neuropeptide precursors is associated with ciliary bands, suggesting potential roles for the neuropeptides derived from these precursors in control of larval locomotion and/or feeding. In conclusion, our findings provide novel perspectives on the evolution and development of neuropeptide signaling systems and neuroanatomical insights into neuropeptide function in echinoderm larvae.

## Introduction

Neuropeptides are neuronal signaling molecules that act as neurotransmitters, neuromodulators, and/or neurohormones to regulate diverse physiological processes and behaviors (Strand, 1999; Veenstra, 2011). They range in size from 3 to over 40 amino acid residues but are derived from larger precursor proteins and are often subject to post-translational modifications during their biosynthesis (e.g., C-terminal amidation; Sossin et al., 1989; Zhou et al., 1999). Investigation of the phylogenetic distribution and relationships of neuropeptide signaling systems has revealed that the evolutionary origin of many neuropeptides can be traced to the common ancestor of the Bilateria (Jékely, 2013; Mirabeau and Joly, 2013). Thus, orthologs of vertebrate neuropeptides have been identified in deuterostomian and protostomian invertebrates (Hewes and Taghert, 2001; Veenstra, 2010, 2011; Semmens et al., 2016). Peptidergic signaling systems have also been identified in the nervous systems of the Cnidaria (e.g., sea anemones), which are a sister group to the Bilateria (Galliot and Quiquand, 2011), and the origins of some peptide signaling systems may even predate the evolution of nervous systems (Schuchert, 1993; Jékely, 2013; Nikitin, 2015).

Many animals have complex life cycles, with larval stage(s) preceding a metamorphic transition to the reproductively active adult stage. However, with the exception of *Drosophila melanogaster* and other insects (Nässel and Winther, 2010), investigation of the physiological roles of neuropeptides has largely focused on reproductively mature adult animals. Thus, little is known about neuropeptide function during the larval stage(s) of most invertebrates. We present below an overview of some of the studies that have analyzed larval neuropeptide systems in a variety of invertebrates from several phyla.

Starting with the Cnidaria, the anatomical distribution of neuropeptides immunoreactive with antibodies to Arg-Phe-NH_2_ (RFamide) has been investigated extensively in the planula larvae of a variety of species (Gröger and Schmid, 2001; Katsukura et al., 2003; Yuan et al., 2008; Marlow et al., 2009; Mayorova and Kosevich, 2013). Interestingly, large numbers of immunoreactive neurons are detected at the anterior pole of the planulae just prior to metamorphosis, indicating that RFamide-type neuropeptides may be involved in larval settlement and metamorphosis. Another class of neuropeptides, the LWamides, have been localized in hydrozoan larvae and found to be involved in control of planula migration and metamorphosis (Leitz et al., 1994; Katsukura et al., 2004; Piraino et al., 2011).

Moving on to the Bilateria, research on lophotrochozoan protostomian invertebrates has, until recently, largely focused on the larvae of species belonging to the phylum Mollusca. For example, neurons expressing small cardioactive peptide (SCP) and FMRFamide were mapped during embryonic and larval development of the gastropods *Tritonia diomedea* and *Haliotis rufescens*. In *T. diomedea*, a role for SCP in regulation of larval feeding behavior was proposed (Kempf et al., 1987), whilst in *H. rufescens* a correlation between expression of SCP and initiation of metamorphosis suggested that SCP-immunoreactive neurons may participate in perception of external metamorphic cues (Barlow and Truman, 1992). Analysis of neuropeptide precursor transcript sequence data has revealed that in *H. rufescens* FMRFamide-related peptides are encoded by two transcripts that appear to be splice variants of a single gene and the two transcript types are expressed in partially overlapping populations of neurons in the larval nervous system (Cummins et al., 2011). The apical organ of many mollusc trochophores is characterized by strong FMRFamide immunoreactivity (ir) (Croll and Voronezhskaya, 1996; Dickinson et al., 1999, 2000; Voronezhskaya et al., 2008) and in larvae of the gastropod *Ilyanassa obsolete* FMRFamide causes muscular contractions and arrest of ciliary beating (Dickinson and Croll, 2003; Braubach et al., 2006). Accompanied by serotonin, FMRFamide is also associated with muscle innervation in trochophores of the bivalve mollusc *Mytilus trossulus* (Dyachuk and Odintsova, 2009). Use of antibodies to other neuropeptide antigens (GWamide, RYamide, and FVamide) has enabled visualization of a variety of neuropeptidergic systems in the veliger larvae of the molluscs *Pecten maximus* (Bivalvia) and *Phestilla sibogae* (Gastropoda; Conzelmann and Jékely, 2012). Furthermore, analysis of the anatomy of neuropeptide systems in molluscan larvae has been extended beyond neuropeptides and neuropeptide precursors to the enzymes involved in neuropeptide biosynthesis (Cummins et al., 2009).

Another lophotrochozoan phylum that has become a focus for research on larval neuropeptide systems is the Annelida, in particular the marine annelids *Platynereis dumerilii* and *Capitella* sp. The nervous systems of the trochophore larvae of these species contain sub-populations of neurons that are immunoreactive with antibodies to DLamide, FVamide, FLamide, GWamide, and RYamide. The specificity of these antibodies has been demonstrated by analysis of the expression of corresponding precursors using mRNA *in situ* hybridization (Conzelmann and Jékely, 2012). Furthermore, in *P. dumerilii* trochophores it has been found that neuropeptides affect ciliary beating, with some causing upward swimming (RYamide, FVMamide, DLamide, FMRFamide, FVamide, LYamide, YFamide, L11, and SPY) and others causing downward swimming (FLamide and WLD; Conzelmann et al., 2011). Experimental analysis of another neuropeptide, myoinhibitory peptide (MIP), revealed that its physiological role changes during the larval development of *P. dumerilii*—inducing settlement in early larvae and regulating feeding and digestion in the later feeding larval stage (Conzelmann et al., 2013; Williams et al., 2015).

Turning to deuterostomian marine invertebrates, sequencing of the genome of the urochordate *Ciona intestinalis* (Dehal et al., 2002) has enabled a comprehensive analysis of larval neuropeptide expression in this species. The expression patterns of genes encoding gonadotropin-releasing hormone (GnRH)-type, oxytocin/vasopressin-type, tachykinin-type, and galanin-type peptides have been mapped and they suggest that these neuropeptides play roles in regulation of osmotic pressure, sensing of environmental cues, larval movement, and initiation of metamorphosis (Hamada et al., 2011). More recently, the genome sequences of hemichordate species have been determined (Simakov et al., 2015), but as yet there has been little investigation of neuropeptide systems in these animals. However, there is a history of neuropeptide research on the other ambulacrian phylum—the echinoderms, which are the focus of this study.

The first echinoderm neuropeptides to be sequenced were the SALMFamide neuropeptides S1 and S2, which were both isolated from the starfish species *Asterias rubens* and *A. forbesi* (Elphick et al., 1991). Generation of antibodies to S1 enabled the first insights into the organization of neuropeptidergic systems in echinoderm larvae to be obtained. Thus, S1-like ir was detected in the lateral ganglia and adhesive disk and in neurites underlying ciliary bands in the larvae of *Patiriella regularis* (Byrne et al., 2001; Byrne and Cisternas, 2002), *Pisaster ochraceus* and *A. rubens* (Moss et al., 1994). Extending the analysis to other echinoderms, in the pluteus larvae of the sand dollar *Dendraster excentricus* S1-like ir was observed in the apical and oral ganglia and in a neural plexus innervating the digestive system (Thorndyke et al., 1992). Similarly, immunocytochemical analysis of the pluteus larvae of the sea urchin *Psammechinus miliaris* revealed S1-like ir in the apical ganglion and in nerves associated with the digestive system. In *P. miliaris*, S1 antibodies also label parts of the adult rudiment, whilst nerves underlying the ciliary bands are immunoreactive with antibodies to S2 (Beer et al., 2001).

Opportunities to extend analysis of larval neuropeptide expression in echinoderms beyond the SALMFamides have been provided by sequencing of the genome of the sea urchin *Strongylocentrotus purpuratus* (Sodergren et al., 2006), which enabled identification of genes encoding many neuropeptides and peptide hormone genes in this species (Burke et al., 2006; Rowe and Elphick, 2012). Furthermore, the expression pattern of insulin-like peptides in the digestive system of the pluteus larvae of *S. purpuratus* has been reported, providing evidence of a role in food assimilation (Perillo and Arnone, 2014).

The foundation for the present study was the recent identification of 40 neuropeptide precursor transcript sequences in the starfish *A. rubens* (Semmens et al., 2013, 2016), which represents the most comprehensive resource to date for echinoderm neuropeptide research. These neuropeptide precursors were identified by analysis of the transcriptome of the radial nerve cords from adult starfish. The larvae of *A. rubens* and other starfish have a completely different anatomy and life style to adult animals and therefore one of the aims here was to investigate if neuropeptides expressed in the adult nervous system are also expressed in starfish larvae. A second aim was to gain anatomical insights into the potential functions of neuropeptide systems in echinoderm larvae.

In this the first multi-gene analysis of neuropeptide expression in echinoderm larvae, we used mRNA *in situ* hybridization techniques to analyze the expression of eight neuropeptide genes in *A. rubens* larvae. The eight neuropeptide precursors selected for analysis in this study include: (a) the L-type and F-type SALMFamide neuropeptide precursors, which are precursors of the prototypical SALMFamides S1 and S2, respectively, and 13 related SALMFamide-type neuropeptides, (b) the precursor of a vasopressin/oxytocin-type neuropeptide (“asterotocin”), (c) the precursor of the neuropeptide NGFFYamide, which belongs to a bilaterian family of neuropeptides that includes human neuropeptide-S (NPS) and crustacean cardioactive peptide (CCAP; Semmens et al., 2013, 2015), (d) ArTRHP, a thyrotropin-releasing hormone (TRH)-type neuropeptide precursor, (e) ArGnRHP, the precursor of a gonadotropin-releasing hormone (GnRH)-type neuropeptide, (f) ArCTP, the precursor of a calcitonin-type neuropeptide, and (g) ArCRHP, the precursor of a corticotropin-releasing hormone (CRH)-type neuropeptide.

## Materials and methods

### Cloning of cDNAs encoding *A. rubens* neuropeptide precursors

Total RNA from radial nerve cords dissected from adult *A. rubens* was isolated using the SV Total RNA Isolation System (Promega, Southampton, UK) and then used to synthesize cDNA using the QuantiTect® Reverse Transcription Kit (QIAgen, Manchester, UK). The cDNAs encoding neuropeptide precursors were amplified by PCR using Phusion® or Q5® High-Fidelity DNA Polymerase (NEB, Hitchin, Hertfordshire, UK) and the specific primers listed in Table 1. PCR cycling conditions used varied depending on the primers and size of the amplicon. The size of the amplicon was determined using gel electrophoresis. PCR products were either gel extracted or column purified using the QIAquick® Gel Extraction Kit (QIAgen, Manchester, UK) before being blunt-end cloned into either pBluescript SKII (+; Agilent Technologies, Stockport, Cheshire, UK) or pCR®-Blunt II-TOPO® vector (Thermo Fisher Scientific, Paisley, UK). Plasmids with the correct sized amplicon were then isolated and sequenced (Eurofins Genomics GmbH, Ebersberg, Germany).

**Table 1:** **Sequences of primers used to clone cDNAs encoding *A. rubens* neuropeptide precursors**.

**Precursor**	**Forward primer**	**Reverse primer**	**Amplicon size (bases)**
S1	TAGCTACTTGACACA	ATATGACTAGTTGAGAGAGG	967
S2	GGATCACCTGCTAGTCTTTAGTC	TGCTTATATTTACACACTTTTGCG	1089
Asterotocin	ACACAGTACTACGATCAG	GTCACAAGTGACCATATC	689
NGFFYamide	AGACCTTATAGGCTTAGAG	GTCATCACGATAACACTC	1189
TRH	CGATAAAGCCAAGTCACTCCA	TAATAGCCCCGCAATATCCAG	922
GnRH	AGAGTCACTGGAGTTAAGA	GAACCTGTATCAAGTTGTTC	490
Calcitonin	CAAAGGCAAGGGAAGAGATCT	CTCCCGTTGTTCAATCCTTTC	399
CRH	GTGTGCCCGGCATCAGTT	CTGCCGTGTGGAGTGGTG	853

### Production of digoxigenin-labeled sense and anti-sense probes for *A. rubens* neuropeptide precursor transcripts

Plasmids containing cloned neuropeptide precursor cDNAs were linearized using the restriction endonucleases (NEB, Hitchin, Hertfordshire, UK) listed in Table 2. Linearized plasmids were then purified using a phenol-chloroform/chloroform-isomylalcohol (Sigma-Aldrich Ltd, Gillingham, UK) extraction or the illustra GFX PCR DNA and Gel Band Purification Kit (GE Life Sciences, Amersham, UK). RNA probes were synthesized from linearized plasmids using a digoxigenin (DIG)-labeled nucleotide triphosphate mix (Roche, Mannheim, Germany) supplemented with dithiothreitol (Promega, Southampton, UK), a placental RNase inhibitor (Promega, Southampton, UK) and the RNA polymerases (NEB, Hitchin, Hertfordshire, UK) listed in Table 2. Reaction products were digested with RNase free DNase (NEB, Hitchin, Hertfordshire, UK) to remove template DNA and then stored at −20°C in 25% formamide made up in saline-sodium citrate buffer.

**Table 2:** **Vectors, restriction enzymes, and RNA polymerases used to produce sense and anti-sense probes for *A. rubens* neuropeptide precursor transcripts**.

**Precursor**	**Vector**	**Sense probe**		**Anti-sense probe**	
		**Restriction endonuclease**	**RNA polymerase**	**Restriction endonuclease**	**RNA polymerase**
S1	pBluescript SKII (+)	BamHI	T7	KpnI	T3
S2	pCR®-Blunt II-TOPO®	HindIII	T7	EcoRV	SP6
Asterotocin	pBluescript SKII (+)	SacI	T7	KpnI	T3
NGFFYamide	pBluescript SKII (+)	SacI	T7	KpnI	T3
TRH	pBluescript SKII (+)	SacI	T7	XhoI	T3
GnRH	pBluescript SKII (+)	XhoI	T3	BamHI	T7
Calcitonin	pBluescript SKII (+)	XbaI	T7	XhoI	T3
CRH	pBluescript SKII (+)	XbaI	T7	XhoI	T3

### Collection and fixation of larvae

**Figure 1:**
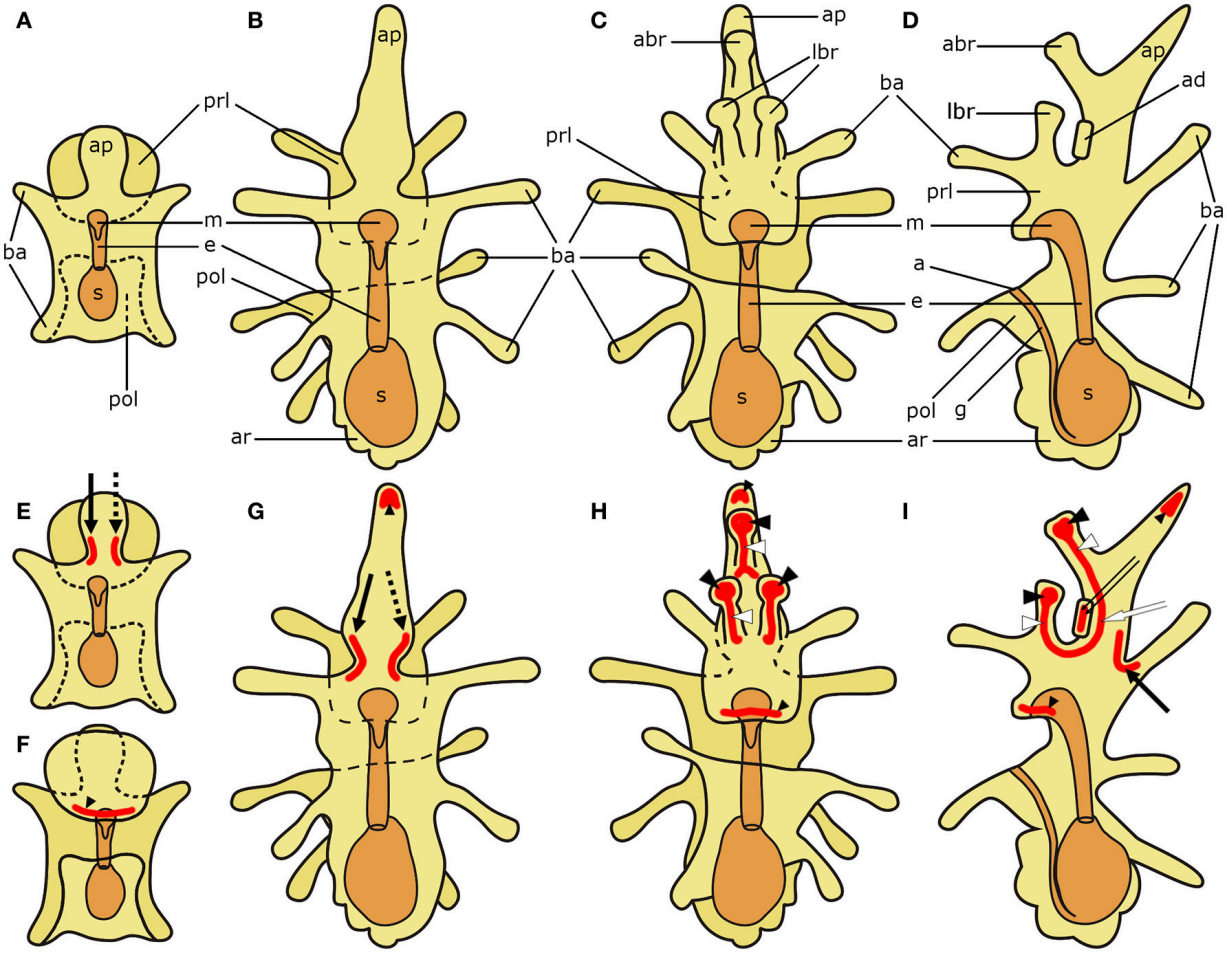
**Schematic diagrams of the general anatomy of starfish (*Asterias rubens*) larvae (modified after, Murabe et al., 2008) (top half) and main neuronal aggregations of the nervous system (bottom half)**. In all diagrams anterior is uppermost with the overall body structure shown in yellow and the digestive system shown in orange. **(A)** bipinnaria larva; dorsal view. **(B–D)** brachiolaria larva. **(B)** dorsal view. **(C)** frontal (ventral) view. **(D)** left lateral view with ventral side on the left and dorsal on the right. **(E)** bipinnaria larva; dorsal view. **(F)** bipinnaria larva; ventral view. (**G–I)** brachiolaria larva. **(G)** dorsal view. **(H)** frontal (ventral) view. **(I)** left lateral view with ventral side on the left and dorsal on the right. The areas of main neuronal aggregations are highlighted in red in **(E–I)**. The lateral ganglia start to develop at the bipinnaria stage and are located at the base of the anterior projection (solid black arrow labels the left lateral ganglion and dotted black arrow labels the right lateral ganglion; Moss et al., 1994; Elia et al., 2009). Neurons are also concentrated in several regions along ciliary bands, especially in the preoral ciliary band near the mouth (small black arrowhead; Moss et al., 1994; Byrne et al., 2007). In addition to these regions, the brachiolaria stage develops neuronal aggregations in the brachia tips (black arrowheads) and stems (white arrowheads), in the adhesive disk (double arrow), and in the basi-epithelial nerve plexus underlying the disk (white arrow; Barker, 1978; Murabe et al., 2008; Elia et al., 2009). a, anus; abr, anterior brachium; ad, adhesive disk; ap, anterior projection; ar, adult rudiment; ba, bipinnaria arms; e, esophagus; g, gut; m, mouth; lbr, lateral brachium; pol, postoral lobe; prl, preoral lobe; s, stomach.

Larvae of the common European starfish *Asterias rubens* Linnaeus, 1758 (Asteroidea, Echinodermata) were collected from plankton samples during the summer seasons of 2014 and 2015 at the Pertsov White Sea Biological Station (Kandalaksha Bay, White Sea, Russia, 66° 34′ N, 33° 08′ E). At the White Sea, the spawning period of *A. rubens* is normally in June so bipinnaria larvae (Figure 1A) and brachiolaria larvae (Figures 1B–D) can be obtained in July and August, respectively. Immediately after collection, larvae were anesthetized with MgCl_2_ and then fixed for 2 h in freshly prepared MEMFA (0.1 M MOPS, pH 7.4, 2 mM EGTA, 1 mM MgSO_4_, and 3.7% formaldehyde). After fixation, larvae were transferred to 100% ethanol and stored at −20°C.

### Whole-mount mRNA *in situ* hybridization

Whole-mount mRNA *in situ* hybridization was performed using a protocol based on methods established for zebrafish larvae (Thisse and Thisse, 2008), including use of sense riboprobes for negative control experiments. In brief, starfish larvae were hybridized with DIG-labeled RNA probes at 65°C overnight. Then they were incubated with alkaline phosphatase-conjugated anti-DIG antibody (Roche, Mannheim, Germany) diluted at 1:5000 at 4°C overnight. Bound antibodies were revealed using nitro blue tetrazolium/5-bromo-4-chloro-3-indolyl phosphate (NBT/BCIP; Roche, Mannheim, Germany) substrate. Experiments in which fixed larvae were just incubated with NBT/BCIP, without probe hybridization and antibody application, were performed as additional control experiments. After incubation in NBT/BCIP, stained larvae were mounted in 90% glycerol and examined under a Leica DM5000 B microscope equipped with a Leica DFC425 C camera (Leica Microsystems GmbH, Wetzlar, Germany). BF and DIC optics were utilized to take images, which were then processed using Adobe Photoshop CS2 and GIMP 2.8.0. The only tools used were brightness/contrast adjustment and brush to clean debris stuck to the surface of specimens.

## Results

### Cloning and sequencing of cDNAs encoding *A. rubens* neuropeptide precursors

The sequences of transcripts encoding *A. rubens* neuropeptide precursors have been identified previously by analysis of radial nerve cord transcriptome sequence data from this species (Semmens et al., 2013, 2016). Here, the expression of eight of these neuropeptide precursors was investigated in *A. rubens* larvae using whole-mount mRNA *in situ* hybridization. To accomplish this it was necessary to clone and sequence cDNAs encoding the neuropeptide precursors. This enabled confirmation of the sequences predicted from assembled transcriptome sequence data and production of templates for generation of DIG-labeled single-stranded RNA probes. The sequence of a cDNA encoding the *A. rubens* L-type SALMFamide (S1) precursor had been cloned and sequenced previously (Jones et al., 2016) and this is shown in Supplementary Figure 1 (GenBank accession number KT601732). The sequences of cDNAs encoding six of the seven other neuropeptide precursors were determined here for the first time, with the exception of the ArGnRH precursor cDNA, which was reported recently (Tian et al., 2016). The sequences of cDNAs encoding the F-type SALMFamide (S2) precursor (Supplementary Figure 2; GenBank accession number KP330476), asterotocin precursor (Supplementary Figure 3; GenBank accession number KT601711), NGFFYamide precursor (Supplementary Figure 4; GenBank accession number KC977457), ArGnRH precursor (Supplementary Figure 6; GenBank accession number KT601712), ArCT precursor (Supplementary Figure 7; GenBank accession number KT601715), and ArCRH precursor (Supplementary Figure 8; GenBank accession number KT601710) were found to be identical to the assembled transcript sequences reported previously (Semmens et al., 2013, 2016). However, sequencing of a cDNA encoding the *A. rubens* TRH-type precursor (ArTRHP) revealed a difference to the predicted transcript sequence reported previously (Semmens et al., 2016). The cloned cDNA comprises a protein-coding region of 768 bases encoding a 256-residue protein (Supplementary Figure 5), which is longer than the 675 base protein-coding region encoding a 225-residue protein in the transcript sequence predicted from assembled radial nerve cord transcriptome sequence data (see Figure S11 in Semmens et al., 2016; GenBank accession number KT601714). Thus, ArTRHP comprises 13 copies of the sequence QWYTG, giving rise to 13 copies of the predicted mature peptide pyroGlu-Trp-Tyr-Thr-NH_2_ (Supplementary Figure 5); this contrasts with the 11 copies of this peptide in the precursor sequence predicted from assembled transcriptome sequence data (Semmens et al., 2016). This discrepancy between the cloned cDNA sequence and the assembled transcript sequence is likely due to an error in the assembly of raw sequence reads arising from the presence of repetitive DNA sequences in this transcript.

### Larval anatomy and analysis of neuropeptide precursor expression using mRNA *in situ* hybridization

To facilitate interpretation of micrographs showing expression of neuropeptide precursors in *A. rubens*, in Figure 1 we show diagrams of the anatomy of the bipinnaria and brachiolaria larvae of this species. The pre-metamorphic stages of development in *A. rubens* are planktonic, with embryonic development finishing at the gastrula stage when the gastrula hatches from a fertilization envelope. The gastrula then elongates, develops ciliary bands, and a mouth opening to form the dipleurula stage. The dipleurula grows to form bipinnaria stage, which is a middle stage of larval development in starfish (Figure 1A). Bipinnariae are oval shaped with two lobes on the ventral side—the preoral lobe anteriorly and the postoral lobe posterior to the mouth. Several short bilaterally symmetrical outgrowths called bipinnaria arms project from the body and the length and number of the arms increase as larvae develop (Figure 1A). A prominent unpaired outgrowth, known as the anterior projection, is located behind the preoral lobe. The bipinnariae are covered with cilia but ciliary coverage is much denser on the rim of the lobes and arms, forming what are referred to as ciliary bands. The preoral lobe overhangs the mouth like a hood. The mouth leads to the esophagus, which runs caudally and enters the stomach. A narrow tubular intestine emerges from the lower part of the stomach, bends toward the ventral side and then runs in an anterior direction until it reaches the anus, which is located on the postoral lobe.

The late larval stage of *A. rubens* is the brachiolaria, which is several times larger than the bipinnaria and bears five pairs of long bipinnaria arms (Figures 1B–D). The digestive system in the brachiolaria larva is similar to the bipinnaria larva. The main difference from the bipinnaria is the presence of specific brachiolar arms, or brachia, on the preoral lobe (Figures 1C,D). In *A. rubens* brachiolaria there are three brachia: an unpaired anterior brachium and a pair of lateral brachia. Each brachium has a thin stem bearing a spherical tip (adhesive papilla). A round adhesive disk is located between the brachia. Collectively, the brachia and the adhesive disk form the attachment complex, which is responsible for attachment prior to metamorphosis of the larva. The rudiment of a future pentaradial starfish (adult rudiment) develops on the left side of the posterior region of the brachiolaria (Figures 1B–D).

In Figures 1E–I, we show (in red) prominent features of the nervous system of starfish larvae that contain aggregations of neuronal cell bodies. These include the lateral ganglia, which start to form during the bipinnaria stage and are located at the base of the anterior projection in brachiolaria larvae (Moss et al., 1994; Elia et al., 2009). Neurons are also concentrated in several regions along ciliary bands, especially in the preoral ciliary band near the mouth (Moss et al., 1994; Byrne et al., 2007). In addition, the brachiolaria stage is characterized by the presence of groups of neurons in the tips and stems of the brachia, in the adhesive disk and in a basi-epithelial nerve plexus underlying the adhesive disk (Barker, 1978; Murabe et al., 2008; Elia et al., 2009).

Use of anti-sense DIG-labeled probes for the eight neuropeptide precursors analyzed here revealed expression in bipinnaria and/or brachiolaria larvae, as described in detail below. In control experiments using sense probes the specific patterns of staining obtained with anti-sense probes were not observed, demonstrating the specificity of the anti-sense probes. However, control experiments in which larvae were incubated with the alkaline phosphatase substrate NBT/BCIP without probes revealed some staining near the mouth, in the lower part of the esophagus and in the stomach in bipinnaria and brachiolaria (not shown). This staining is likely due to the presence of endogenous phosphatases, which are often present in tissues of the digestive system (Yu et al., 2007; Shifrin et al., 2012). However, the intensity of this staining was much weaker in larvae that had been taken through the mRNA *in situ* hybridization protocol, probably due to inactivation of endogenous phosphatases by the high temperatures (65°C) used in this technique (Middleton et al., 2001; Murphy et al., 2001).

### Localization of L-type SALMFamide (S1) precursor transcripts in *A. rubens* larvae

**Figure 2:**
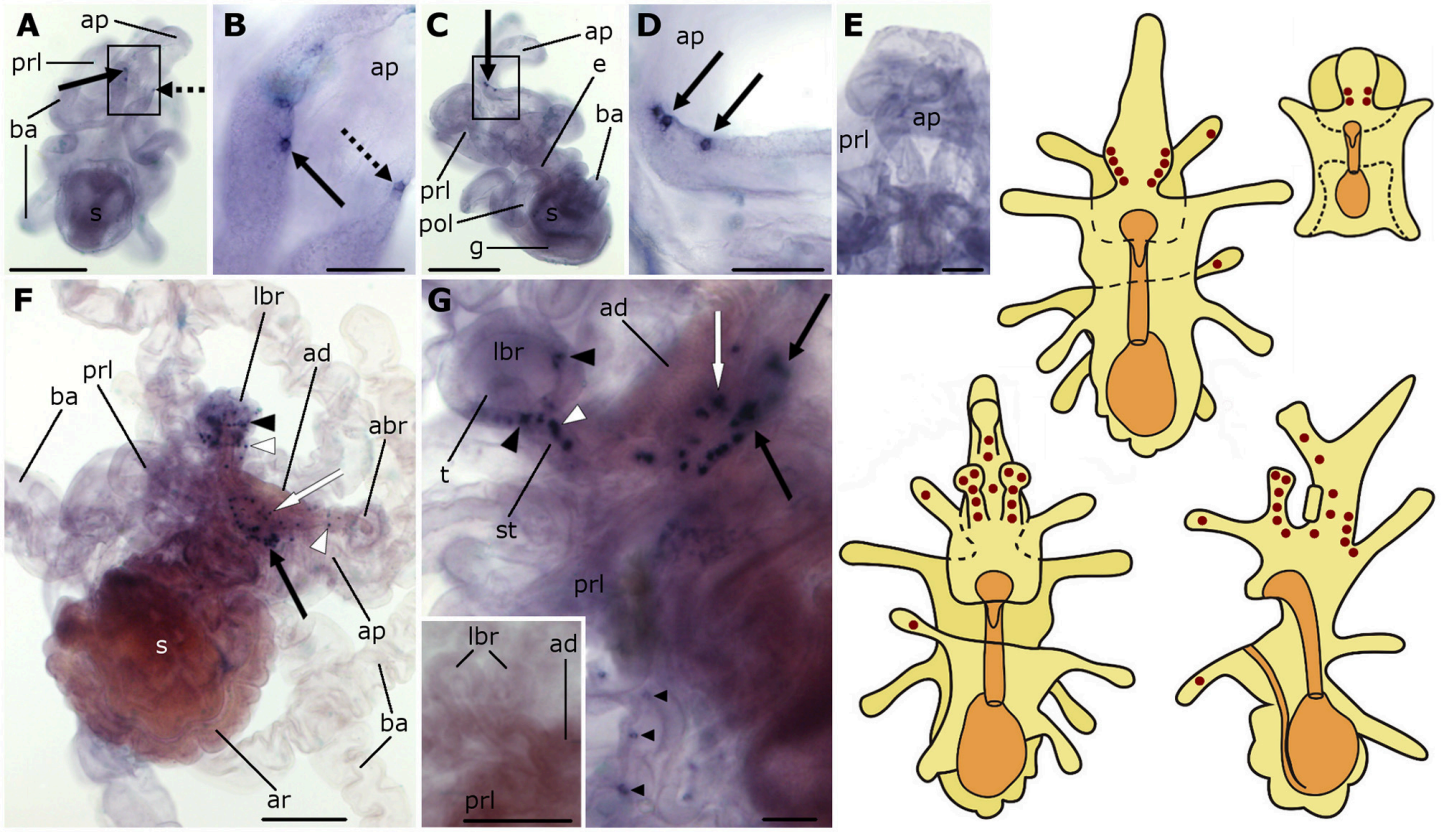
**Localization of L-type SALMFamide (S1) precursor transcripts in larvae of the starfish *Asterias rubens* using whole-mount *in situ* hybridization. (A–E)** bipinnaria larva; **(F,G)** brachiolaria larva; the schematic drawings illustrate the distribution of stained cells (see Figure 1 for labeling). **(A)** dorsal view showing paired groups of 2–3 stained cells on the left (solid arrow) and right (dashed arrow) sides of the anterior projection. **(B)** detail of the anterior projection (boxed region in **A**) showing stained cells on the left and right sides (solid and dashed arrows, respectively). **(C)** left lateral view showing three stained cells at the base of the anterior projection (solid arrow). **(D)** detail of the anterior projection (boxed region in **C**) showing stained cells at the base of the anterior projection (solid arrows). **(E)** dorsal view of the anterior projection showing absence of staining in a larva incubated with sense probes. **(F)** left lateral view showing expression in solitary cells of the brachia (black and white arrowheads) and around the adhesive disk (white arrow), and in a group of tightly packed cells in the dorsal region of the larva (black solid arrow). **(G)** detail of the attachment complex, showing stained cells present in both the tip (black arrowheads) and the stem (white arrowheads) of a lateral brachium. In addition to a group of cells under the adhesive disk (white arrow), there are also scattered solitary stained cells (small black arrowheads) that appear to be located along the ciliary bands of the preoral lobe and a group of stained cells in the dorsal region of the larva (black solid arrows). Inset shows the attachment complex of a brachiolaria larva incubated with sense probe control. abr, anterior brachium; ad, adhesive disk; ap, anterior projection; ar, adult rudiment; ba, bipinnaria arms; e, esophagus; g, gut; lbr, lateral brachium; pol, postoral lobe; prl, preoral lobe; s, stomach; st, stem of brachium; t, tip of brachium. Scale bars: 200 μm (**A,C,F**, inset of **G**), 50 μm **(B,D,E,G)**.

Expression of the L-type SALMFamide (S1) precursor is first observed at the base of the anterior projection in early bipinnaria larvae (Figure 2A). This expression is evident in one or two stained cells located along the rim on each side of the anterior projection (Figure 2B). Then as bipinnariae develop the number of these cells increases to 2–5 cells (Figures 2C,D). This staining is not observed in bipinnariae incubated with sense probes (Figure 2E), demonstrating the specificity of the staining observed with anti-sense probes.

As the brachiolaria forms, strong staining is observed in ~20 cells located in the dorsal domain of the upper body region, which corresponds to the base of the anterior projection (Figure 2F). Stained cells are also observed in brachia and near to the adhesive disk, which itself is unstained (Figures 2F,G). The lateral brachia have many cells expressing the precursor, located both in the stem and tip (Figure 2G). By contrast, the anterior brachium contains fewer stained cells and these are restricted to the stem (Figure 2F). In addition, a few solitary cells expressing the precursor are observed along the bipinnaria arms and at the margin of the preoral lobe in brachiolariae (Figure 2G). The staining observed in brachiolariae with anti-sense probes was not observed in control experiments using sense probes (Figure 2G, inset).

### Localization of F-type SALMFamide (S2) precursor transcripts in *A. rubens* larvae

**Figure 3:**
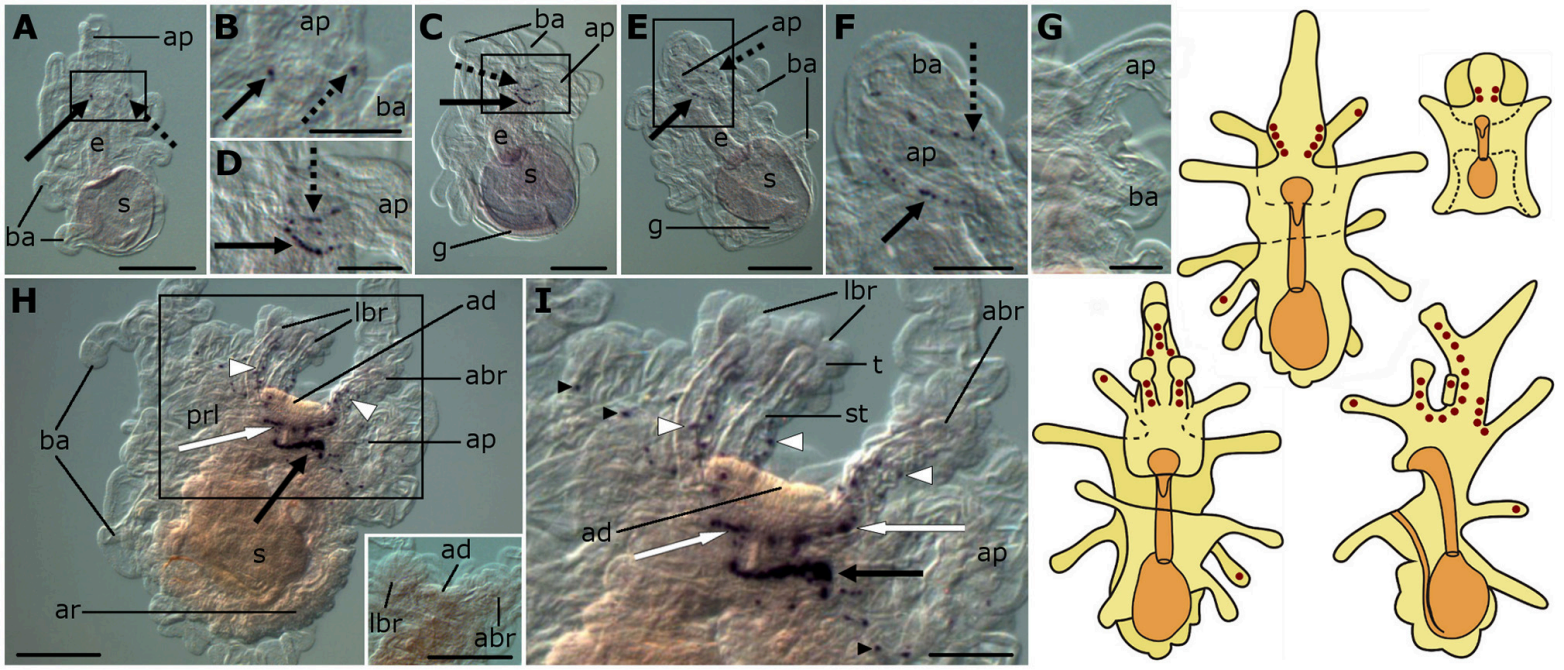
**Localization of F-type SALMFamide (S2) precursor transcripts in larvae of the starfish *Asterias rubens* using whole-mount *in situ* hybridization. (A–G)** bipinnaria larva; **(H,I)** brachiolaria larva; the schematic drawings illustrate the distribution of stained cells (see Figure 1 for labeling). **(A)** early bipinnaria, dorsal view showing stained cells on the left (solid arrow) and right (dashed arrow) sides of the anterior projection. **(B)** detail of the anterior projection (boxed region in **A**) showing stained cells on the left and right sides (solid and dashed arrows, respectively). **(C)** later bipinnaria, left lateral view showing two symmetrical groups of cells along the left (solid arrow) and right (dashed arrow) sides of anterior projection. **(D)** detail of the anterior projection (boxed region in **C**) showing stained cells on the left and right sides (solid and dashed arrows, respectively). **(E)** later bipinnaria, dorsal view showing stained cells along the margin of the anterior projection, with solid and dashed arrows highlighting cells on the left and right side, respectively. **(F)** detail of the anterior projection (boxed region in **E**) showing stained cells on the left and right sides (solid and dashed arrows, respectively). **(G)** left lateral view of anterior projection showing absence of staining in a larva incubated with sense probes. **(H)** left lateral view, showing expression in brachia (white arrowheads), near the adhesive disk (white arrow), and in a ganglion on the dorsal side (black arrow). Inset shows the attachment complex of a brachiolaria larva incubated with sense probe control. **(I)** detail of the attachment complex (boxed region in **H**), showing expression in separate cells along the brachium stems (white arrowheads) and arms (small black arrowhead). Groups of stained cells are located close to the adhesive disk (white arrows) and on the dorsal side (black arrow) of the larva. abr, anterior brachium; ad, adhesive disk; ap, anterior projection; ar, adult rudiment; ba, bipinnaria arms; e, esophagus; g, gut; lbr, lateral brachium; prl, preoral lobe; s, stomach; st, stem of a brachium; t, tip of a brachium. Scale bars: 200 μm (**A,C,E,H**, inset of **H**), 100 μm **(B,D,F,G,I)**.

The expression pattern of the F-type SALMFamide (S2) precursor is similar to that of the L-type SALMFamide (S1) precursor. Expression appears very early during development, localized in two cells on each side of the base of the anterior projection in bipinnaria larvae (Figures 3A,B). As development proceeds, the number of cells in this position increases and staining can be observed in two parallel rows of 10 or more cells located along the rim of the anterior projection (Figures 3C–F). This staining was not observed in control experiments where bipinnariae were incubated with sense probes (Figure 3G).

The pattern of expression expands significantly at the brachiolaria stage. Very strong staining is observed on the dorsal side of the larva in the lower region of the anterior projection, associated with a dense population of up to 30 cells on each side (Figure 3H). In addition to these ganglion-like structures, stained cells are scattered along the stems of the brachia but these do not extend into the tips of the brachia. Approximately 15 stained cells underlie the adhesive disk (Figures 3H,I) but the adhesive disk itself is sparsely stained, with just one or two solitary cells expressing the precursor. Stained cells are also located along the rim of the anterior projection and in the bipinnaria arms of brachiolariae (Figures 3H,I). None of the brachiolaria staining with anti-sense probes was observed in control experiments with sense probes (Figure 3H, inset).

### Localization of asterotocin precursor transcripts in *A. rubens* larvae

**Figure 4:**
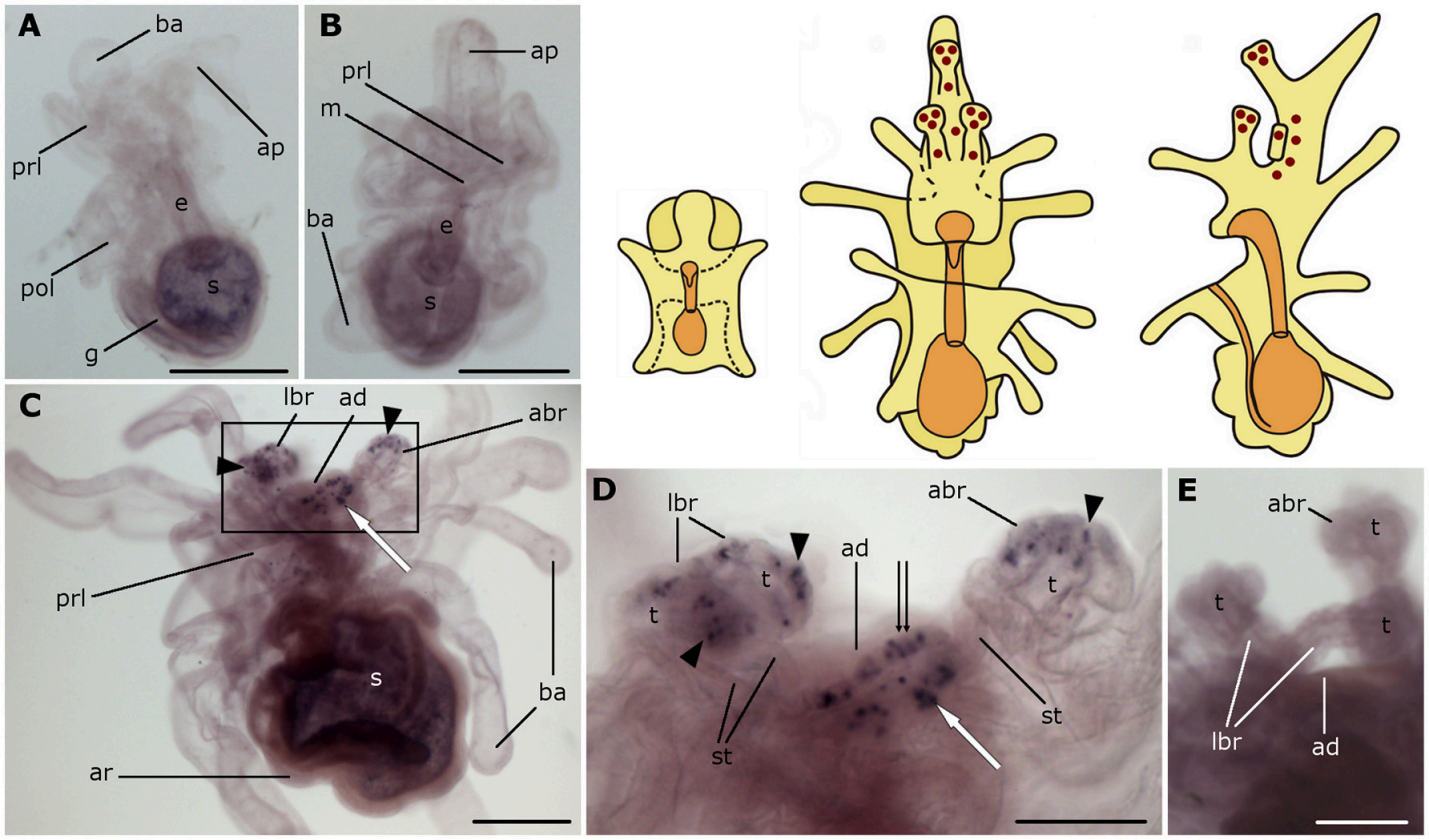
**Localization of asterotocin precursor transcripts in larvae of the starfish *Asterias rubens* using whole-mount *in situ* hybridization. (A,B)** bipinnaria larvae, showing no detectable expression of asterotocin precursor transcripts (**A**, left lateral view; **B**, frontal view). **(C–E)** brachiolaria larva. The schematic drawings illustrate the distribution of stained cells (see Figure 1 for labeling). **(C)** left lateral view, showing expression in the tips of the brachia (black arrowheads) and near the adhesive disk (white arrow). **(D)** detail of the attachment complex (boxed region in **C**) showing stained cells in the brachium tips (black arrowheads), the adhesive disk (double arrow), and adjacent to the adhesive disk (white arrow). **(E)** detail of the attachment complex showing absence of staining in a larva incubated with sense probes. abr, anterior brachium; ad, adhesive disk; ap, anterior projection; ar, adult rudiment; ba, bipinnaria arms; e, esophagus; g, gut; m, mouth; lbr, lateral brachium; pol, postoral lobe; prl, preoral lobe; s, stomach; st, stem of a brachium; t, tip of a brachium. Scale bars: 200 μm **(A–C)**, 100 μm **(D,E)**.

Expression of the asterotocin precursor is not observed in bipinnaria larvae (Figures 4A,B). However, prominent expression is observed in mature brachiolaria larvae, in association with the attachment complex and adjacent tissues. The tips of all three brachia contain multiple stained small cells located in the epithelium (Figures 4C,D), whereas the stems of the brachia are void of staining. Several cells in the adhesive disk as well as a group of ~15 cells near the adhesive disk exhibited expression (Figures 4C,D). No staining was observed in the attachment complex (Figure 4E) or other brachiolaria structures in control experiments using sense probes.

### Localization of NGFFYamide precursor transcripts in *A. rubens* larvae

**Figure 5:**
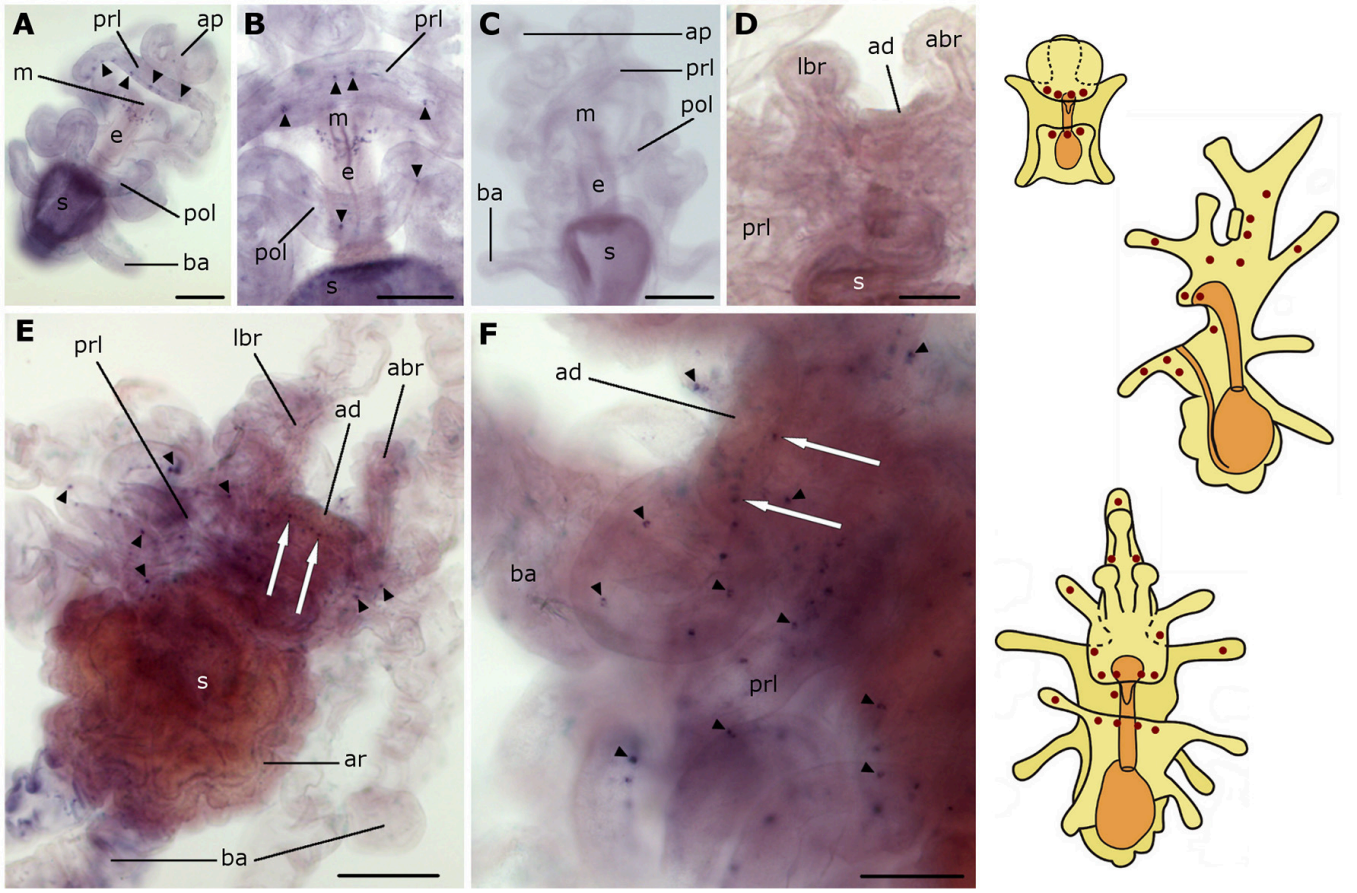
**Localization of NGFFYamide precursor transcripts in larvae of the starfish *Asterias rubens* using whole-mount *in situ* hybridization. (A–C)** bipinnaria larva; **(D–F)** brachiolaria larva; the schematic drawings illustrate the distribution of stained cells (see Figure 1 for labeling). **(A)** frontal view, showing solitary stained cells along the ciliary band of the preoral lobe (small arrowheads). **(B)** detail of the mouth region, showing expression in solitary cells (small arrowheads) along the ciliary bands of both preoral and postoral lobes. **(C)** detail of the mouth region showing absence of staining in a larva incubated with sense probes. **(D)** middle part of the body and the attachment complex, showing absence of staining with sense probes. **(E)** left lateral view, showing stained cells scattered throughout the larval body (small arrowheads) and under the adhesive disk (white arrows). **(F)** attachment complex and the middle part of the body, showing stained cells along the rims (small arrowheads) and under the adhesive disk (white arrows). abr, anterior brachium; ad, adhesive disk; ap, anterior projection; ar, adult rudiment; ba, bipinnaria arms; e, esophagus; m, mouth; lbr, lateral brachium; pol, postoral lobe; prl, preoral lobe; s, stomach. Scale bars: 100 μm **(A–D,F)**, 200 μm **(E)**.

Expression of the NGFFYamide precursor first appears in early bipinnariae, with staining located in solitary cells along the frontal rim of the preoral lobe (Figure 5A). Later, bipinnariae exhibit expression in cells scattered along the frontal rims of both the preoral and the postoral lobes (Figure 5B). In control experiments with sense probes stained cells were not observed (Figures 5C,D).

In brachiolariae, cells expressing the NGFFYamide precursor are scattered throughout the larval body, but predominately in the upper region (Figures 5E,F). These cells are located in the epithelium of the preoral lobe and in proximal regions of the bipinnaria arms, whereas distal parts of the arms do not exhibit expression. The stained cells are localized at the margin of the preoral lobe (Figure 5F) and several stained cells are located underneath the adhesive disk (Figures 5E,F). However, no expression was observed in the adhesive disk itself or in the brachia (Figures 5E,F).

### Localization of ArTRHP transcripts in *A. rubens* larvae

**Figure 6:**
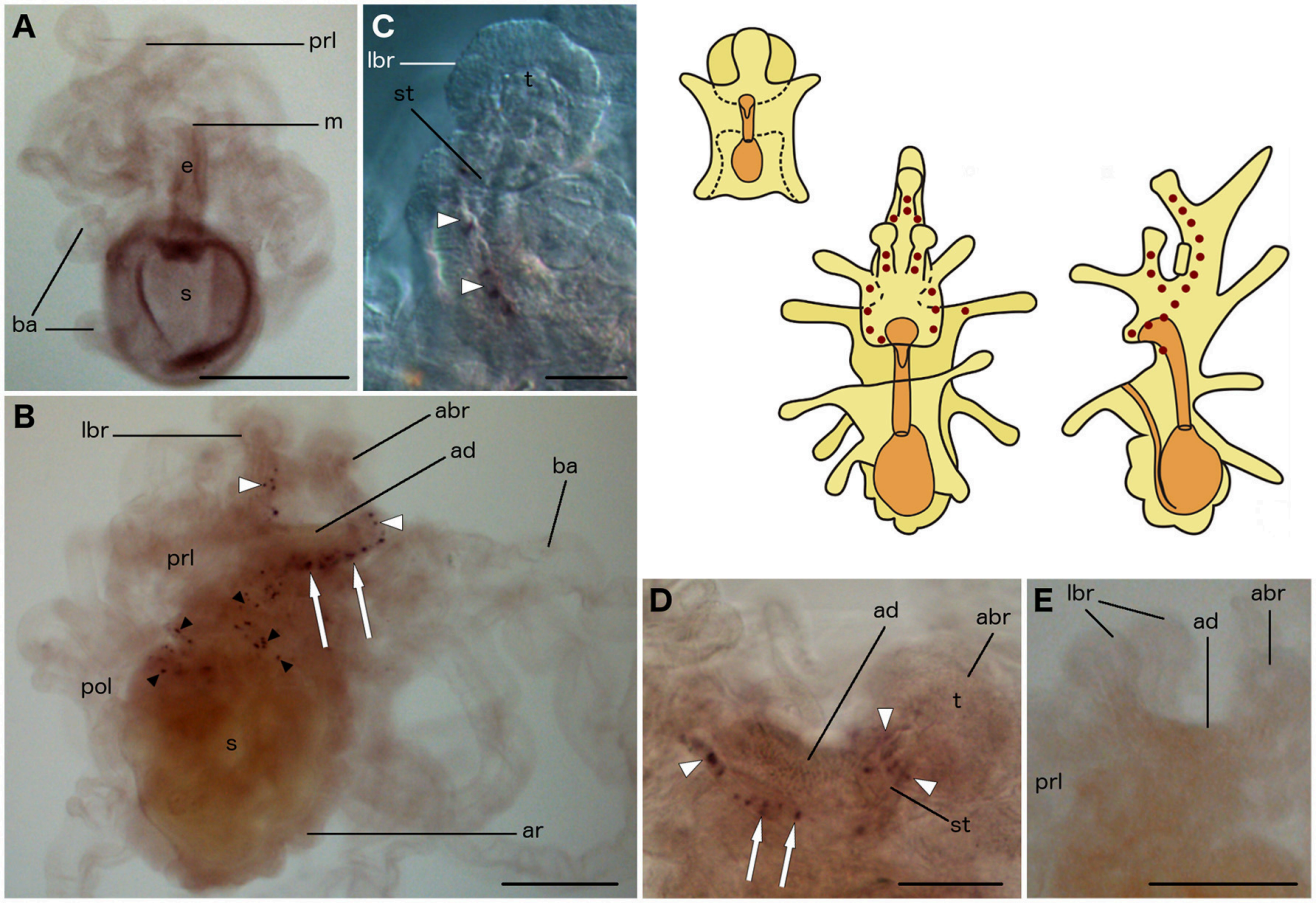
**Localization of ArTRHP transcripts in larvae of the starfish *Asterias rubens* using whole-mount *in situ* hybridization. (A)** bipinnaria larva, frontal view showing no detectable expression of ArTRHP transcripts; **(B–E)** brachiolaria larva; the schematic drawings illustrate the distribution of stained cells (see Figure 1 for labeling). **(B)** left lateral view, showing stained cells along the brachium stems (white arrowheads), near the adhesive disk (white arrows) and throughout the preoral lobe (small black arrowheads). **(C)** detail of a lateral brachium; note the presence of stained cells (white arrowheads) in the stem, but not in the tip of the brachium. **(D)** detail of the attachment complex; stained cells are present in the brachium stem (white arrowheads) and near the adhesive disk (white arrows). **(E)** attachment complex and the middle part of the body, showing no staining with sense probes. abr, anterior brachium; ad, adhesive disk; ar, adult rudiment; ba, bipinnaria arms; e, esophagus; m, mouth; lbr, lateral brachium; pol, postoral lobe; prl, preoral lobe; s, stomach; st, stem of a brachium; t, tip of a brachium. Scale bars: 200 μm **(A,B,E)**, 50 μm **(C,D)**.

No expression of ArTRHP was observed in bipinnariae (Figure 6A). However, prominent expression occurs in mature brachiolariae, where stained cells can be observed in the brachia, around the adhesive disk and, more sparsely, in the preoral lobe (Figure 6B). The stained cells in the brachia are located in the stem epithelium, but no expression is evident in the tips (Figures 6B–D). A group of about 10 cells expressing ArTRHP are located close to the adhesive disk, but no expression is evident in cells of the adhesive disk itself (Figures 6B,D). A row of loosely packed stained cells runs from the attachment complex along the preoral lobe (Figure 6B) and this staining is not observed in brachiolariae incubated with sense probes (Figure 6E).

### Localization of ArGnRHP transcripts in *A. rubens* larvae

**Figure 7:**
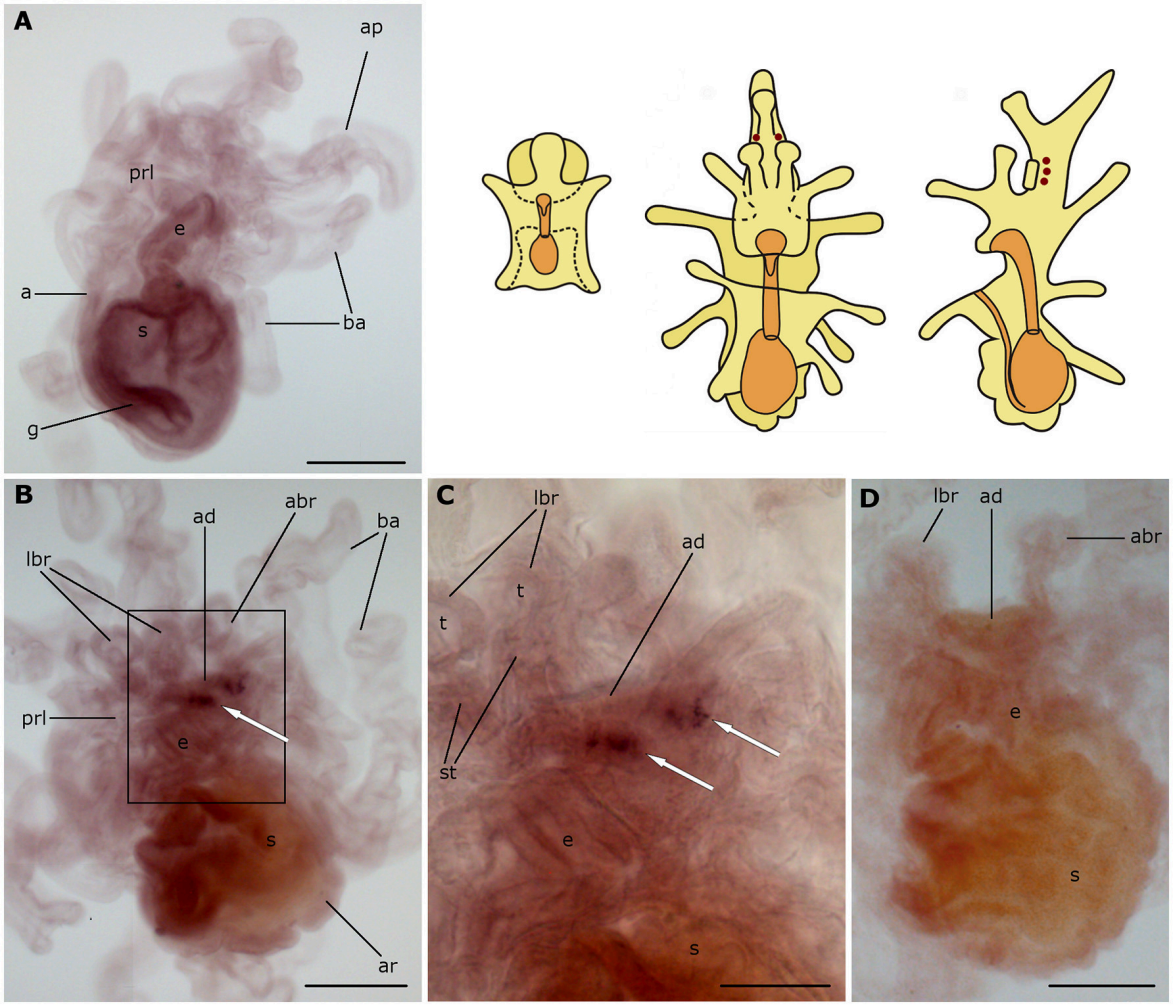
**Localization of ArGnRHP transcripts in larvae of the starfish *Asterias rubens* using whole-mount *in situ* hybridization. (A)** bipinnaria larva, left lateral view, showing no detectable expression of ArGnRHP transcripts; **(B–D)** brachiolaria larva; the schematic drawings illustrate the distribution of stained cells (see Figure 1 for labeling). **(B)** left lateral view, showing stained cells close to the adhesive disk (white arrow) **(C)** detail of the attachment complex (boxed region in **B**) showing two groups of stained cells near the adhesive disk (white arrows). **(D)** absence of staining in a brachiolaria larva incubated with sense probes. a, anus; abr, anterior brachium; ad, adhesive disk; ap, anterior projection; ar, adult rudiment; ba, bipinnaria arms; e, esophagus; g, gut; lbr, lateral brachium; prl, preoral lobe; s, stomach; st, stem of a brachium; t, tip of a brachium. Scale bars: 200 μm **(A,B,D)**, 100 μm **(C)**.

No expression of ArGnRHP was observed in bipinnariae (Figure 7A). In brachiolariae, ArGnRHP expression is limited to a group of cells near the adhesive disk (Figures 7B,C). These cells are grouped as two ganglion-like structures, with 5–10 cells in each group, located on each side of the adhesive disk near to the anterior brachium. No such staining was evident in brachiolariae incubated with sense probes (Figure 7D).

### Localization of ArCTP transcripts in *A. rubens* larvae

**Figure 8:**
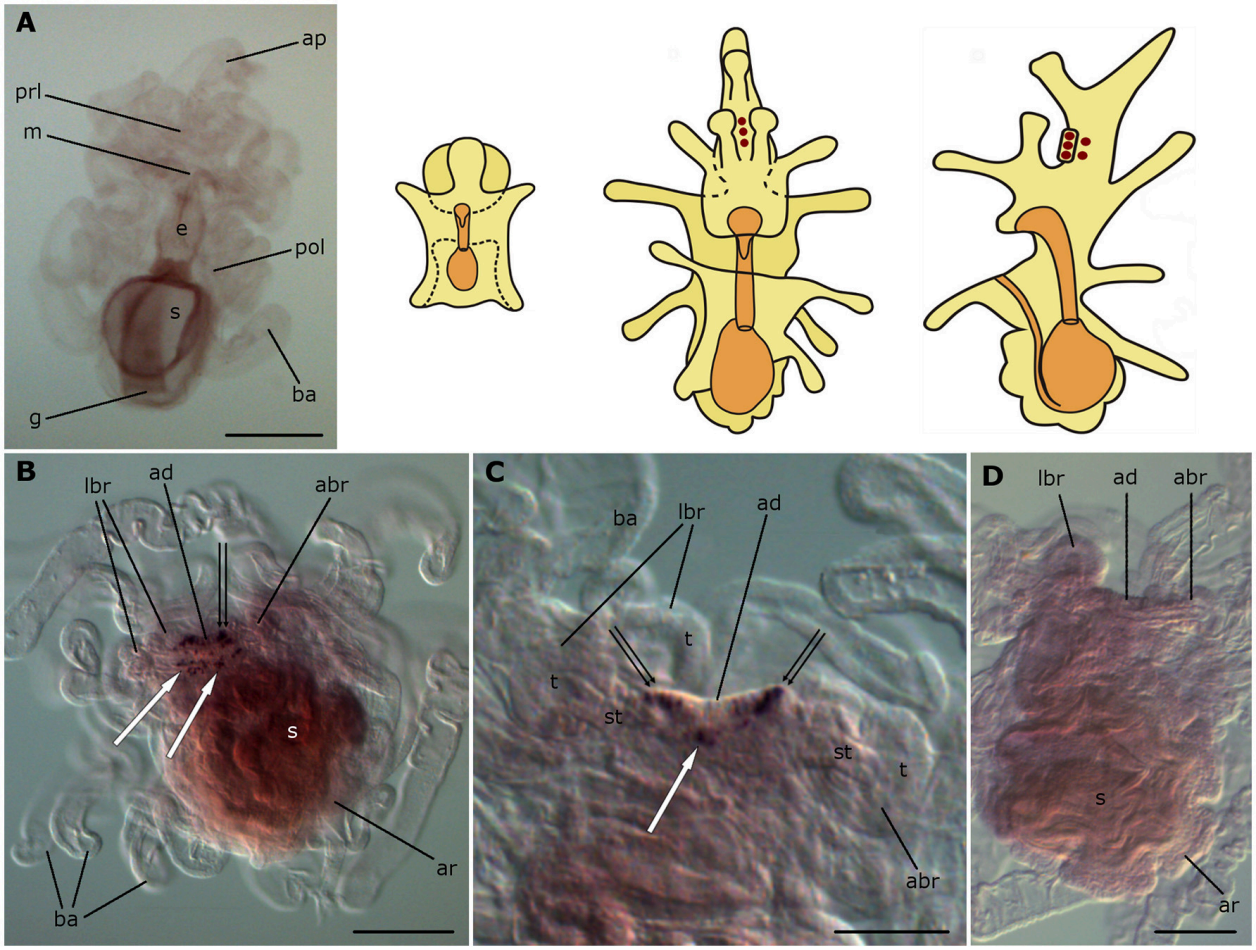
**Localization of ArCTP transcripts in larvae of the starfish *Asterias rubens* using whole-mount *in situ* hybridization. (A)** bipinnaria larva, frontal view, showing no detectable expression of ArCTP transcripts; **(B–D)** brachiolaria larva; the schematic drawings illustrate the distribution of stained cells (see Figure 1 for labeling). **(B)** left lateral view, showing stained cells in the adhesive disk (double arrow) and adjacent to the adhesive disk (white arrows). **(C)** detail of the attachment complex; note stained cells within the adhesive disk (double arrows) and adjacent to it (white arrow). **(D)** absence of staining in a brachiolaria larva incubated with sense probes. abr, anterior brachium; ad, adhesive disk; ap, anterior projection; ar, adult rudiment; ba, bipinnaria arms; e, esophagus; g, gut; m, mouth; lbr, lateral brachium; pol, postoral lobe; prl, preoral lobe; s, stomach; st, stem of a brachium; t, tip of a brachium. Scale bars: 200 μm **(A,B,D)**, 100 μm **(C)**.

No expression of ArCTP was observed in bipinnariae (Figure 8A), whereas in brachiolariae there is expression in the adhesive disk and adjacent tissue (Figure 8B). The stained cells in the disk are located peripherally and have a characteristic columnar shape that can be distinguished from other epithelial cells (Figure 8C). In addition, up to 30 cells expressing ArCTP are located in adjacent tissue surrounding the disk (Figures 8B,C). This staining was not observed in control experiments where brachiolariae were incubated with sense probes (Figure 8D).

### Localization of ArCRHP transcripts in *A. rubens* larvae

**Figure 9:**
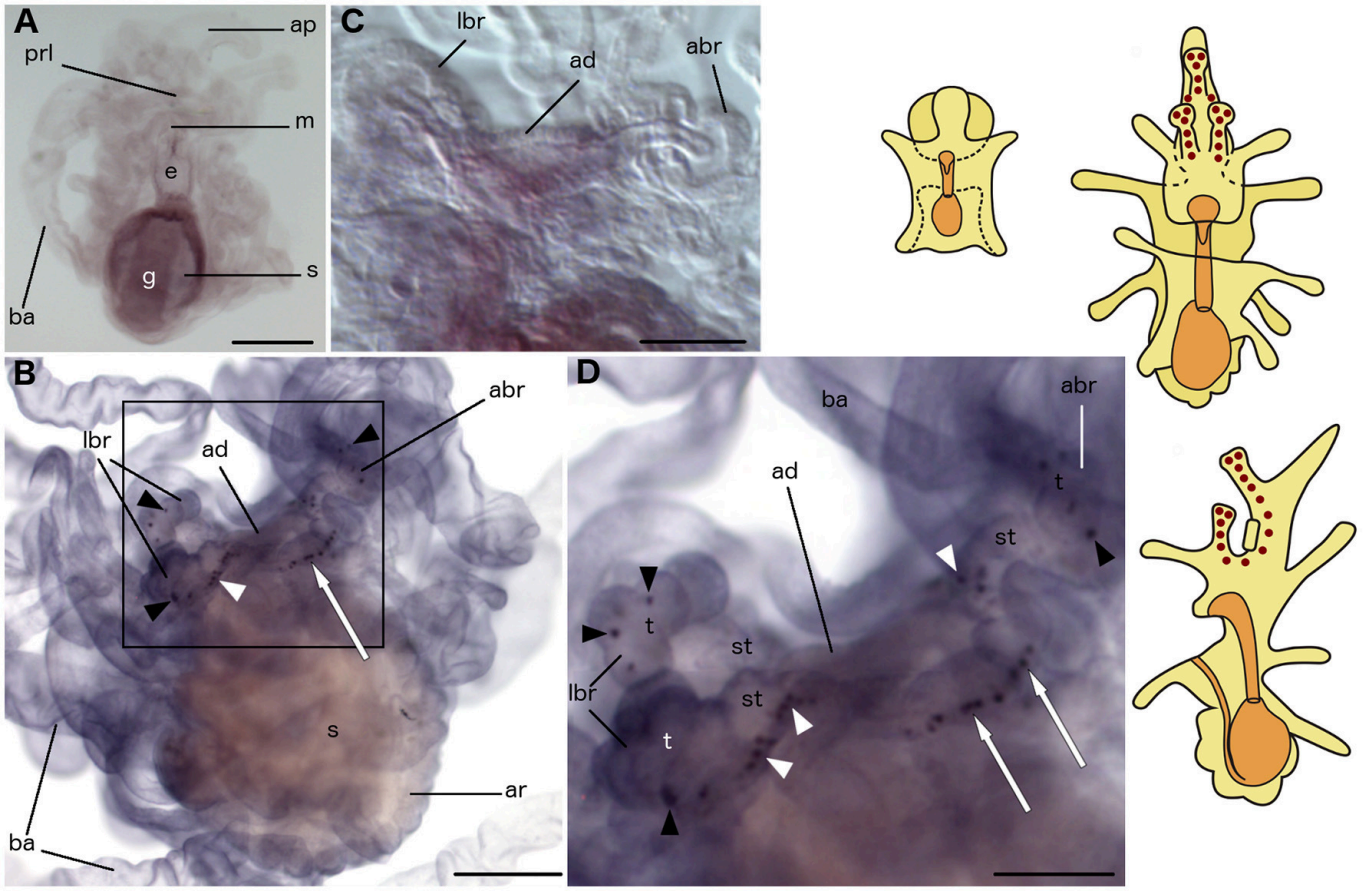
**Localization of ArCRHP transcripts in larvae of the starfish *Asterias rubens* using whole-mount *in situ* hybridization. (A)** bipinnaria larva, frontal view, showing no detectable expression of ArCRHP transcripts; **(B–D)** brachiolaria larva; the schematic drawings illustrate the distribution of stained cells (see Figure 1 for labeling). **(B)** left lateral view, showing stained cells along the brachia (black and white arrowheads) and near the adhesive disk (white arrow). **(C)** detail of the attachment complex showing absence of staining in a larva incubated with sense probes. **(D)** detail of the attachment complex (boxed region in **B**) showing stained cells in tips (black arrowheads) and stems (white arrowheads) of brachia and close to the adhesive disk (white arrows). abr, anterior brachium; ad, adhesive disk; ap, anterior projection; ar, adult rudiment; ba, bipinnaria arms; e, esophagus; g, gut; m, mouth; lbr, lateral brachium; prl, preoral lobe; s, stomach; st, stem of a brachium; t, tip of a brachium. Scale bars: 200 μm **(A,B)**, 100 μm **(C,D)**.

No expression of ArCRHP was observed in bipinnariae (Figure 9A), whereas in brachiolariae there is expression in the brachia and in tissues adjacent to the adhesive disk (Figure 9B). The expression pattern consists of two rows of small cells, with each row starting at the tip of the lateral brachium, extending along the stem of the lateral brachium and then runs around the adhesive disk before extending up the stem of the anterior brachium to its tip (Figures 9B,D). Thus, the rows of stained cells extending from the two lateral brachia fuse together in the stem of the anterior brachium, with ~50 cells in total expressing ArCRHP. The specificity of this staining was confirmed by an absence of staining in experiments using sense probes (Figure 9C).

## Discussion

Here, we report the first multi-gene analysis of neuropeptide precursor expression in larvae of an echinoderm—the starfish *Asterias rubens*. Employing use of mRNA *in situ* hybridization methods, we have investigated larval expression of eight neuropeptide precursors, which were first identified as transcripts in the nervous system (radial nerve cords) of adult *A. rubens* (Semmens et al., 2016). Expression of all eight precursors analyzed was detected in mature brachiolaria larvae and expression of three of the precursors (L- and F-type SALMFamide; NGFFYamide) was also observed earlier in bipinnaria larvae.

The anatomy of starfish larvae (and other echinoderms) is dramatically different to that of adult animals. For example, the larvae are bilaterally symmetrical whereas the adult animals typically exhibit pentaradial symmetry. Furthermore, echinoderm larvae undergo a catastrophic metamorphosis during which the starfish juvenile grows within and then consumes larval tissues (Oguro et al., 1976; McEdward and Miner, 2001; Haesaerts et al., 2005; Murabe et al., 2007; Morris et al., 2011). The larval nervous system disintegrates during metamorphosis, so larval neurons are not incorporated into the adult body (Chia and Burke, 1978). Furthermore, neurogenesis in the larval and adult nervous systems of starfish may be controlled by different transcription factors (Byrne et al., 2005). In this context, it is interesting that the same neuropeptide signaling systems are expressed in both the larval and adult starfish nervous systems. However, the physiological roles of these neuropeptide systems may change during larval development and in the transition to an adult nervous system. Consistent with this notion, functional analysis of myoinhibitory peptide (MIP) in the annelid *Platynereis dumerilii* has revealed that this neuropeptide induces settlement in the early larvae but in late larvae it is involved in feeding behavior and digestion (Conzelmann et al., 2013; Williams et al., 2015).

Anatomical mapping of the expression of neuropeptide genes in starfish larvae, as reported here, provides a basis for investigation of neuropeptide function during starfish larval development and comparison with physiological roles in adult animals. With this long term objective in mind, we discuss below the functional significance of the neuropeptide gene expression patterns reported here, and their relationship with different parts of the larval nervous system, which are as follows. At the bipinnaria stage, the main neuronal accumulations are found at the base of the anterior projection (lateral ganglia) and in the frontal part of preoral lobe (Figures 1E,F; Moss et al., 1994; Elia et al., 2009). The nervous system of brachiolaria includes the lateral ganglia in the anterior projection and neuron accumulations located within the attachment complex, and ciliary bands (Figures 1G–I; Burke, 1983; Nakajima et al., 2004; Elia et al., 2009). There are also loose neuronal meshworks innervating the body wall and digestive system in brachiolaria (Murabe et al., 2008). With the exception of the latter, expression of at least two neuropeptide genes was found to be associated with these known components of the starfish larval nervous system, as discussed below. It is likely, therefore, that most or all of the cells visualized here as expressing neuropeptide genes are neurons. However, we cannot exclude the possibility that some of the cells expressing neuropeptide genes are non-neuronal cell types (e.g., endocrine cells).

### SALMFamide neuropeptide precursor expression in lateral ganglia

The SALMFamides are a family of neuropeptides that act as muscle relaxants in adult echinoderms (Elphick, 2014). The prototypes for this neuropeptide family, S1 and S2, were both isolated from extracts of *A. rubens* radial nerve cords (Elphick et al., 1991). S1 is derived from an L-type SALMFamide precursor protein, which comprises other L-type SALMFamides that have the C-terminal motif Leu-X-Phe-NH_2_ (where X is variable). In contrast S2, which is also an L-type SALMFamide, is derived from an F-type SALMFamide precursor that is largely comprised of F-type SALMFamides with the C-terminal motif Phe-X-Phe-NH_2_ (Semmens et al., 2016).

Expression of the S1 and S2 precursors is first observed at the bipinnaria stage in cells located at the base of the anterior projection. During bipinnaria growth, the number of cells expressing S1 and S2 precursors in this region increases and by the brachiolaria stage bilaterally symmetrical tight clusters of stained cells can be seen at the base of the anterior projection. This location most likely corresponds to the lateral ganglia, which normally differentiate in early bipinnaria (Murabe et al., 2008; Elia et al., 2009). Accordingly, S1 precursor mRNA *in situ* hybridization data presented here is consistent with immunocytochemical studies that revealed S1-immunoreactive cells in the lateral ganglia of larvae of several asteroid species (Moss et al., 1994; Byrne and Cisternas, 2002).

The lateral ganglia in starfish larvae are considered to be parts of the apical organ, which is a conserved but morphologically varied feature of larval echinoderms (Byrne et al., 2007). Furthermore, the presence of serotonergic neurons has been found to be a universal characteristic of echinoderm apical organs (Byrne et al., 2007). Interestingly, in addition to aforementioned studies on starfish larvae (Moss et al., 1994; Byrne and Cisternas, 2002), immunocytochemical studies using antibodies to S1 have revealed immunoreactive cells in the apical ganglion of the sand dollar *Dendraster excentricus* and the sea urchin *Psammechinus miliaris* (Thorndyke et al., 1992; Beer et al., 2001). Therefore, the presence of SALMFamide-expressing cells may likewise be a conserved feature of the apical organs in larval echinoderms. Opportunities to further investigate this issue have been provided recently with the identification of genes/transcripts encoding SALMFamide precursors in species from all five of the extant echinoderm classes (Elphick et al., 2015).

### Mosaic expression patterns of neuropeptide precursors in the attachment complex

The attachment complex of brachiolariae comprises the brachia and adhesive disk. It has been reported previously that multiple neurons are associated with the brachia and the adhesive disk and the processes of these neurons form a basi-epithelial nerve plexus that underlies the entire attachment complex (Barker, 1978; Murabe et al., 2007; Elia et al., 2009). Here we have identified neuropeptide precursors that are expressed in the attachment complex and, intriguingly, they have distinctive patterns of expression. Some precursors are expressed in the adhesive disk but not in brachia (ArCTP), whilst others are expressed in the brachia tips but not in the stems (asterotocin) or *vice versa* (ArTRHP; Table 3). These distinct patterns of expression presumably reflect different physiological roles of these neuropeptides in mediating the process of attachment.

**Table 3:** **Comparative expression patterns of neuropeptide precursors in *A. rubens* brachiolaria, in association with known structures of the brachiolaria nervous system**.

**Neuropeptide precursor**	**Lateral ganglia**	**Brachia**		**Adhesive disk**	**Basi-epithelial nerve plexus under adhesive disk**	**Ciliary bands, body wall**
		**Tip**	**Stem**			
S1	+	+/−	+	−	+	+
S2	+	−	+	+	+	+
Asterotocin	−	+	−	+	+	−
NGFFYamide	−	−	−	−	+	+
TRH	−	−	+	−	+	+
GnRH	−	−	−	−	+	−
Calcitonin	−	−	−	+	+	−
CRH	−	+	+	−	+	−

*+, expression registered; −, no expression; +/−, S1 precursor is expressed in the tips of only lateral brachia*.

In mature brachiolaria, the tips of the brachia sense environmental cues for temporary contact with the substrate, whereas the adhesive disk provides permanent adhesion when a substrate suitable for metamorphosis is detected (Haesaerts et al., 2005; Murabe et al., 2007). Furthermore, different types of secretory cells enable attachment and detachment of brachia and these cells are regulated by neurosecretory-like cells (Haesaerts et al., 2005). Thus, neuropeptides expressed in the brachia tips (asterotocin) and the adhesive disk (ArCT), may be involved in control of temporary or permanent attachment, respectively (Table 3). Neuropeptides expressed in the stems of the brachia (S2 and ArTRH) may participate in neural mechanisms of signal transmission and processing. Neuropeptides expressed throughout the brachia and basi-epithelial nerve plexus (S1 and ArCRH) may be involved in perception of environmental cues and/or coordination of attachment complex activity (Table 3). They may also link environmental cues to behavior. Clearly, predictions of function based solely on cellular patterns of mRNA expression is speculative but further insights may be obtained if the axon projections of neuropeptidergic cells are revealed using antibodies specific for neuropeptides that are expressed in the attachment complex.

All of the neuropeptide precursors analyzed in this study were found to be expressed by cells located near the adhesive disk in an area corresponding to the basi-epithelial nerve plexus (Elia et al., 2009; Table 3). This indicates that this region comprises a diverse population of neuropeptidergic cells. Several of these neuropeptide precursors are orthologs of neuropeptide precursors that are expressed in the vertebrate hypothalamus—e.g., vasopressin and oxytocin, GnRH, TRH, and CRH (Okon and Koch, 1976; Sawchenko et al., 1984). Interestingly, in the invertebrate chordate *Ciona intestinalis* orthologs of GnRH and vasopressin/oxytocin are also co-expressed in an area of the nervous system considered to be homologous to the hypothalamus of vertebrates (Hamada et al., 2011). Accordingly, the neuropeptide expression profile of a region of the starfish larval nervous system near the adhesive disk may likewise be reflective of homology with the hypothalamus.

### Neuropeptide expression along the lobe rims and bipinnaria arms—regulators of ciliary beating?

Bands of cilia located along the rims of the larval lobes and along the arms generate water currents necessary for larval locomotion and transfer of food particles to the mouth. Dense accumulations of neurons are present along the rims of the lobes and these are probably involved in regulation of ciliary beating (Moss et al., 1994; Chee and Byrne, 1999; Murabe et al., 2008; Elia et al., 2009). Previous immunocytochemical studies revealed that S1-immunoreactive neurons are associated with the ciliary bands (Moss et al., 1994) and consistent with these findings here we have revealed the presence of the S1 precursor transcripts in a few solitary cells along the bipinnaria arms and at the margin of the preoral lobe in brachiolariae. Furthermore, we have identified several precursors of other neuropeptides that are expressed by cells associated with the ciliary bands, including S2, NGFFYamide and ArTRH (Table 3). The expression of the NGFFYamide precursor is noteworthy because it is already evident soon after a larva becomes able to feed, at the bipinnaria stage, where it is expressed in cells along the ciliary bands near the mouth opening. For this reason, NGFFYamide is a good candidate as a regulator of swimming and/or feeding behavior in *A. rubens* larvae. Interestingly, NGFFYamide triggers contraction and retraction of the cardiac stomach in adult starfish, indicating a role in terminating the extra-oral feeding behavior of adult starfish (Semmens et al., 2013). Thus, NGFFYamide may be involved in regulation of feeding behavior throughout the starfish lifecycle, regulating ciliary beating around the mouth in larvae and triggering cardiac stomach retraction in adults.

Brachiolariae exhibit much more complex locomotor behavior than bipinnaria larvae (Haesaerts et al., 2001, 2005) and therefore regulation of this activity may require recruitment of neurochemical control systems. This may explain why additional cells expressing different neuropeptides (S1, S2, and ArTRH) differentiate along the ciliary bands at the brachiolaria stage.

### Comparison of the expression patterns of evolutionarily related neuropeptides

Six of the eight neuropeptide precursors analyzed in this study can be grouped into three pairs of evolutionarily related proteins:

Firstly, the L-type and F-type SALMFamide precursors, which may have arisen by duplication of a common ancestral gene in an ancestor of the eleutherozoan echinoderms (Asterozoa and Echinozoa; Elphick et al., 2015), which is estimated to have diverged from the lineage that gave rise to extant crinoids ~500 million years ago (Pisani et al., 2012). In this context, it is interesting that the expression patterns of the L-type and F-type SALMFamide precursors in starfish larvae are similar (Table 3). It remains to be determined if the two SALMFamide precursors are co-expressed in the same cells, or in partially overlapping populations of cells or in mutually exclusive populations of cells. Nevertheless, the similarity in their expression patterns presumably reflects their evolutionary origins as paralogs in eleutherozoan echinoderms. For this reason it would be interesting to investigate the larval expression patterns of the single SALMFamide-type precursor that has been identified in species belonging to the basal class of extant echinoderms—the crinoids (Elphick et al., 2015).

Secondly, the asterotocin and NGFFYamide precursors are paralogs that originated by gene duplication in a common ancestor of the Bilateria (Urbilateria; Semmens et al., 2015). Thus, the gene duplication that ultimately gave rise to the asterotocin and NGFFYamide precursors occurred earlier (probably >550 million years ago; Erwin and Davidson, 2002) than the gene duplication that gave rise to the L-type and F-type SALMFamide precursors in eleutherozoan echinoderms. This is interesting because unlike the two SALMFamide precursors, the expression patterns of the asterotocin and NGFFYamide precursors are quite different. This may reflect a more ancient divergence in the physiological roles of asterotocin and NGFFYamide as paralogous neuropeptides.

Thirdly, the ArCT and ArCRH precursors are evolutionarily related members of a bilaterian families of neuropeptides that act on the secretin-type class of G-protein coupled receptors. Thus, as with asterotocin and NGFFYamide, the evolutionary origins of ArCT and ArCRH can be traced back to Urbilateria. Little is known about the physiological roles of CRH-type and CT-type neuropeptides in invertebrates, with the notable exception of insects, where both neuropeptides act as diuretic hormones (Kataoka et al., 1989; Furuya et al., 2000). In this context, it is interesting that ArCTP and ArCRHP are both expressed in the attachment organs of *A. rubens* brachiolariae, but with non-overlapping patterns of expression—ArCTP is expressed in the attachment disk, whereas ArCRH is expressed in the brachia. This suggests that these peptides may have distinct but related roles associated with larval attachment.

## General conclusions and perspectives

This is the first study to map the expression of neuropeptide precursor genes in larvae of an echinoderm. By mapping the expression of eight neuropeptide precursors in *A. rubens* larvae we have obtained new insights into the complex neurochemical architecture of larval echinoderm nervous systems. On-going studies are investigating the expression patterns of multiple neuropeptide precursors in adult *A. rubens* and this will provide opportunities to make comparisons with the larval expression patterns reported here.

A detailed anatomical and pharmacological analysis of neuropeptide signaling has been accomplished using the free swimming larvae of the marine annelid *P. dumerilii* as a protostomian invertebrate model system (Conzelmann et al., 2011; Williams et al., 2015). This has revealed the roles of specific neuropeptides in regulation of processes such as settlement, feeding, and ciliary beating. Accordingly, pharmacological approaches could now be employed to investigate and compare the functions of neuropeptides in the larvae of deuterostomian invertebrates, including echinoderms such as the starfish *A. rubens*.

## Author contributions

All authors had full access to all the data in the study and take responsibility for the integrity of the data and the accuracy of the data analysis. Study concept and design: TM and MRE. Acquisition of data: TM (material collection, ISH); ST (cloning and synthesis of probes for ArTRHP and ArGnRHP); WC (cloning of ArCRH and probe synthesis for ArCRHP and ArCTP); DS and EO (cloning and synthesis of probes for asterotocin and NGFFYamide precursors); MZ (cloning and synthesis of probes for S2 precursor); YB (cloning of ArCTP), MR and ME (cloning and probe synthesis for S1 precursor). Analysis and interpretation of data: TM and MRE. Drafting of the manuscript: TM, DS, and MRE. Obtained funding: TM, MRE. Technical and material support: ME. Study supervision: MRE.

## Funding

This study was supported by a European Molecular Biology Organization fellowship awarded to TM (ASTF 168-2014), Russian Foundation for Basic Research (grant 15-04-04298 awarded to TM), BBSRC (grant BB/M001644/1), the Leverhulme Trust (grant RGP-2013-351), the China Scholarship Council and the Society for Experimental Biology.

### Conflict of interest statement

The authors declare that the research was conducted in the absence of any commercial or financial relationships that could be construed as a potential conflict of interest.
