# Supplementary material for: Localization of Neuropeptide Gene Expression in Larvae of an Echinoderm, the Starfish Asterias rubens
Source: Front Neurosci. 2016 Dec 1;10:553. doi: 10.3389/fnins.2016.00553 (PMC5130983; doi:10.3389/fnins.2016.00553)
Supplement: Supplementary file 1 [file DataSheet1.DOCX]

Supplementary Material

**Localization of neuropeptide gene expression in larvae**

**of an echinoderm, the starfish *Asterias rubens***

**Tatiana D. Mayorova, Shi Tian, Weigang Cai, Dean C. Semmens, Esther A. Odekunle, Meet Zandawala, Yusef Badi, Matthew L. Rowe, Michaela Egertová, Maurice R. Elphick^*^**

***Correspondence:** Prof. Maurice R. Elphick: [m.r.elphick@qmul.ac.uk](mailto:m.r.elphick@qmul.ac.uk)

1 ***t***

2 ***agctacttgacacatacac****actgcgtaacactcgggtcgcaagctacttctttgctcgct*

62 *ggccgtagacaactctttgatatctctcgaccaccatcaaaaacgcagacacaacacaat*

122  *ttcgcgtggtttatttaaacgcacacgaggggcctacctgccttgggttttttccgggtg*

182 *tttaattttgtgtactcaccgtcccaaatgaagggccaacaccttctagccgtagcagtg*

***M K G Q H L L A V A V***  11

242 *gttgtcgtcgccggttcgtttggaatcatcgaggcgtactctccatttgggggctataac*

***V V V A G S F G I I E A*** ***Y S P F G G Y N*** 31

302 *cgagcaccttttgacaatgtttgggtgcgagcggacagcatggctcgtggaggctcgacg*

***R A P F D N V W V R A D S M A R G G S T*** 51

362 ggggaggacgaagccaacgaacagcgaatgacgggagccaagcgaccggctgg*t*gcctca

***G E D E A N E Q R M T G A K R P A G A S*** 71

422 *gcgttccactccgctctgtcgtacggcaaacgaggcgacgatgacagcgccgaagtggag*

***A F H S A L S Y G K R G D D D S A E V E***  91

482 *cgccgagcctaccactcggccctgcccttcggcaagagaacacccatcgagaaacgcgcc*

***R R A Y H S A L P F G K R T P I E K R A*** 111

542  *taccacacaggtctccccttcggaaagagggacgacgaagccgccgaacaagacgccatg*

***Y H T G L P F G K R D D E A A E Q D A M*** 131

602 *atggagaggcgtggcttcaactcggccctgatgttcggtaaacgactacacagtgctcta*

***M E R R G F N S A L M F G K R L H S A L*** 151

662 *ccgttcggtaagcgcggctaccacagtgctctgccgttcgggaagagattggataccact*

***P F G K R G Y H S A L P F G K R L D T T*** 171

722 *gatgaaggagat*atcatcgagagaagaggttaccatagcgggctaccgttcggcaagcgc

***D E G D* I I E R R G Y H S G L P F G K R** 191

782 gctactgacgatgaagccgttaatgacatactagaccaattaagaagcgaagagaattga

**A T D D E A V N D I L D Q L R S E E N** * 210

842 cttttataaagaactgtaatccttaaaaatcagcttatatactaatctaacagtgcttag

902 gatacgacaatatcggttggtgatcagtttagttaccttacaaaac**cctctctcaactag**

962 **tcatat**

**Supplementary Figure 1. *A. rubens* L-type SALMFamide (S1) precursor cDNA sequence.** The nucleotide sequence (lowercase, 967 bases) encoding the precursor protein (uppercase, 210 amino acid residues) is shown. Primers used for cloning are represented in bold and underlined text. The predicted signal peptide is represented in blue, seven putative L-type SALMFamide peptides are represented in red, C-terminal glycine (G) residues that are known or predicted substrates for amidation are represented in orange and putative dibasic cleavage sites are represented in green. The asterisk shows the position of the stop codon. Note that cloning and sequencing has revealed changes on the nucleotide level, which are represented in italic and underlined text. This sequence has been deposited in the GenBank database and assigned the accession number KT601732, which has been confirmed by cloning and sequencing. Also note that probes were generated to a partial region of the L-type SALMFamide precursor (due to the restriction endonuclease EcoRV cutting within the precursor), with this partial region highlighted in italic text.

1 **gg**

3 **atcacctgctagtctttagtc**tcgctggaataaaagtcttcaacatctaaacgcagacga

63 gtaaaggtatctttttttactttactcttccgtattctcagataacgacgggaaaaagtc

123 aagataatttcacgtacacaactcttcagaagaagaacggaagcaagaaaggataagatg

**M**  1

183 atggtgcgattcgtagccttactcggggcagtcagcctactggtatgtcaatctgcagga

**M V R F V A L L G A V S L L V C Q S A G**  21

243 cttgatgccgcagacgtcgaggaacaagacgagttcaacaaaccctatgctcctgacagt

**L D A A D V E E Q D E F N K P Y A P D S** 41

303 tcgtatgcggatttaaatgcacttttgggcaacaatgtgccaagtctacacagcgcctcc

**S Y A D L N A L L G N N V P S L H S A S** 61

363 aagcgtcaacaaagtgacagggagcgtgaggttgaagcagcccagacgcaattttacccg

**K R Q Q S D R E R E V E A A Q T Q F Y P** 81

423 tatggaagaagaactgatcccaggaaagcgtctggtggattcacctttggcaagagaggg

**Y G R R T D P R K A S G G F T F G K R G** 101

483 cagtattttatccccattccgtacgagaaacgagagatggatgaggtgaacccgtacagc

**Q Y F I P I P Y E K R E M D E V N P Y S** 121

543 gtagctaagcgcgacgacgagctgaccggactagaggagtaccaagctagcaagaggtca

**V A K R D D E L T G L E E Y Q A S K R S** 141

603 ggtccttattcctttaacagcgggctgacctttggcaagagggaacccgagaagaggaac

**G P Y S F N S G L T F G K R E P E K R N** 161

663 atattcggatcttatgacttcgggaagcgggcttacggcaacaatttcagcttcggcaag

**I F G S Y D F G K R A Y G N N F S F G K** 181

723 cgaggcatgggagtgtccagttttagctttggcaaacgatccggacttgagggtgaacaa

**R G M G V S S F S F G K R S G L E G E Q** 201

783 atgatgccggaagacaaacgggcgttcggagacttttccttcggcaagcgcaataatggt

**M M P E D K R A F G D F S F G K R N N G** 221

843 ctgtccagcttcacattcggcaagcgagagggtgaacgatagaacacgagagggcgccat

**L S S F T F G K R E G E R *** 234

903 actgtctacaatgtgataattatagtatcttaattatttcaaaaccatacttgataagaa

963 ataactgcttgcgttttgagttaaaacatcagttccaagtatacaaacaaattttaaata

1023 acattgtttaaagcacggattttgaattaaaatgaaacgataa**cgcaaaagtgtgtaaat**

1083 **ataagca**

**Supplementary Figure 2. *A. rubens* F-type SALMFamide (S2) precursor cDNA sequence.** The nucleotide sequence (lowercase, 1089 bases) encoding the precursor protein (uppercase, 234 amino acid residues) is shown. Primers used for cloning are represented in bold and underlined text. The predicted signal peptide is represented in blue, seven putative F-type SALMFamide peptides and the L-type SALMFamide S2 are represented in red, C-terminal glycine (G) residues that are known or predicted substrates for amidation are represented in orange and putative dibasic cleavage sites are represented in green. The asterisk shows the position of the stop codon. This cDNA sequence is identical to part of a longer assembled transcript sequence, which has been deposited in the GenBank database under accession number KP330476.

1 **ac**

3 **acagtactacgatcag**cagacgagattgagggacgatcgctggtttttttgcgagagtac

63 gcgattgataacgacttcatcatcaattggctcagatacgtttattgaataacctgttgt

123 gaagagaaaagttgtccaaggatattccacagaacttccgtccggtggacgatgggcatg

**M G M**  3

183 aagtcaatggtggcgctgtggactggggtactggtcgcactgtgggttcaaagtcaagca

**K S M V A L W T G V L V A L W V Q S Q A**  23

243 tgtttagttcaagactgtccggaaggaggaaagaggtccagttataacacaatcagacag

**C L V Q D C P E G G K R S S Y N T I R Q** 43

303 tgcctatcctgcggccctggtggtttgggacaatgtgtaggttcagctatatgctgcggc

**C L S C G P G G L G Q C V G S A I C C G** 63

363 aatactttcggctgttttctcgggacaaaagaaaccttcgtgtgcagagaagaaagtcag

**N T F G C F L G T K E T F V C R E E S Q** 83

423 ctctccacaccttgtgaggttgttggagagacatgtgaatctattactgacgggaaatgt

**L S T P C E V V G E T C E S I T D G K C** 103

483 gtttcaaacggcttctgttgcaatgagagaagctgctctttagacgtagcgtgcagagaa

**V S N G F C C N E R S C S L D V A C R E** 123

543 accgatacagaacagagagacctcaaaaacagactcaaagagaggcttctggacgccctc

**T D T E Q R D L K N R L K E R L L D A L** 143

603 ttgcgtcaaccatgaacccagttaccactccctcaactttgttgataattttcacccaca

**L R Q P *** 147

663 atgcaatgt**gatatggtcacttgtgac**

**Supplementary Figure 3. *A. rubens* asterotocin precursor cDNA sequence.** The nucleotide sequence (lowercase, 689 bases) encoding the precursor protein (uppercase, 147 amino acid residues) is shown. Primers used for cloning are represented in bold and underlined text. The predicted signal peptide is represented in blue, the putative asterotocin peptide is represented in red, a C-terminal glycine (G) residue that is a putative substrate for amidation is represented in orange and a putative dibasic cleavage site is represented in green. The C-terminal region of the precursor comprises a neurophysin domain (purple; with the fourteen cysteine (C) residues that are a characteristic and conserved feature of neurophysins underlined). The asterisk shows the position of the stop codon. This cDNA sequence is identical to part of a longer assembled transcript sequence, which has been deposited in the GenBank database under accession number KT601711.

1 **ag**

3 **accttataggcttagag**aggaccatcgagaagagcttgagttactttacctggcgctcag

63 gtgggaattcattttctatcagcaagaacactccttagtttacaatcaattacaagtgga

123 atatcgctcatttggaaacatcaacaagattttgacgaactaggaggggtgtcggtggga

183 cgtgggggatctaagctggatatgaccatgggcagcaggtcgttattagtgacaattgtg

**M T M G S R S L L V T I V** 13

243 atcacagtagtcatacccagcatctgggcaggtgcaatagctggggctcaaacacaaaag

**I T V V I P S I W A G A I A G A Q T Q K** 33

303 attcgtcgtgaaagtcgagaatctggcaagtactggccaaactccgtgggtatctcagac

**I R R E S R E S G K Y W P N S V G I S D** 53

363 caacagctacggcaactcctagcacactctctggcggactcgtacagtacgtcaggggca

**Q Q L R Q L L A H S L A D S Y S T S G A**  73

423 agtcacatacggggaggagacggggatgcagggtatatatacgatagtcgagatcaggtc

**S H I R G G D G D A G Y I Y D S R D Q V** 93

483 gatgacacggggacgaacgaggaggaaggggaacgcgtaatcgggagcgaggttacatcg

**D D T G T N E E E G E R V I G S E V T S** 113

543 agagactcgaaccccggtacaagcaagagaaatgggttcttctatggcaaaagaaatggg

**R D S N P G T S K R N G F F Y G K R N G** 133

603 ttcttttatggaaagagatcagcgtcaacccctggcaatgcaaatgaagtaactcaatgc

**F F Y G K R S A S T P G N A N E V T Q C** 153

663 atcccgtgtgggcctcaaaacaacggccagtgcgtcatgtttggtacatgttgcagctat

**I P C G P Q N N G Q C V M F G T C C S Y** 173

723 gaactaggtggctgctttttcctgacagaggaggcccttccctgtgtgacgtcaaaatcg

**E L G G C F F L T E E A L P C V T S K S** 193

783 tcatcattatgtgagctgagcggattgccgtgcggtgacgagggatatggaaggtgcgtg

**S S L C E L S G L P C G D E G Y G R C V** 213

843 gcagactctgtctgttgtctgccgcaagagggctcttgtcatattaacgcagaatgtgga

**A D S V C C L P Q E G S C H I N A E C G** 233

901 ggcaagatgacatttcaataggacttgcattatgcggactttaaattatttataaaggga

**G K M T F Q *** 239

963 taggaaaaggtggttaatatctgtattttgaaaagggtaataaaatttaaggttgtttga

1023 gaaaagggacacgaatgttattttgacctcaatgtgtaaatttaaacaattttagcgatt

1083 acttatttttagaccactacgaattaactgttttatgttctttacgacgggaaagaaatg

1143 aaatcttttgagagattcgaatgtacaaa**gagtgttatcgtgatgac**

**Supplementary Figure 4. *A. rubens* NGFFYamide precursor cDNA sequence.** The nucleotide sequence (lowercase, 1189 bases) encoding the precursor protein (uppercase, 239 amino acid residues) is shown. Primers used for cloning are represented in bold and underlined text. The predicted signal peptide is represented in blue, two copies of the neuropeptide NGFFYamide are represented in red but with the C-terminal glycine (G) residue that is a substrate for amidation represented in orange and putative dibasic cleavage sites are represented in green. The C-terminal region of the precursor comprises a neurophysin domain (purple; with the fourteen cysteine (C) residues that are a characteristic and conserved feature of neurophysins underlined). The asterisk shows the position of the stop codon. This cDNA sequence is identical to part of an assembled transcript sequence, which has been deposited in the GenBank database under accession number KC977457.

1 **cgataaagccaagtcactcca**cggcaagggacgagcaccctgttattttgacccgggccg

61 gtaggccgttagcgtccagtcactataatcatgtcggttacgagaaacagcggttttcta

**M S V T R N S G F L** 10

121 cttgttactctactgttcacttgggtggtttgtcgcgctgagttggcagatttcgtggag

**L V T L L F T W V V C R A E L A D F V E**  30

181 aatgctgaggtggcgaaagaagtatctaatgaaattg*cag*aaggagtagaagcagagcaa

**N A E V A K E V S N E I *A* E G V E A E Q**  50

241 tggcagagggacgaagacaa*g*agacaatacccaggaggggctcccattggtctagatggt

**W Q R D E D K R Q Y P G G A P I G L D G** 70

301 aaaagacaatggtacaccggcaagcggcaatggtatacgggtaaacgggacgctgaagac

**K R Q W Y T G K R Q W Y T G K R D A E D** 90

361 tcgcccgcattattagcagaaaacgataaacgccaatggtacactggcaaacggagcggc

**S P A L L A E N D K R Q W Y T G K R S G** 110

421 aacgaagaacagcagcccgacgaggcgaacaagagacagtggtataccggcaaacggcaa

**N E E Q Q P D E A N K R Q W Y T G K R Q** 130

481 tggtacaccggcaagcgaggggacgaagacagggtgctggacgacgatgccgtgaactct

**W Y T G K R G D E D R V L D D D A V N S**  150

541 ctcaaacgccagtggtacaccggaaaacgccagtggtacaccggtaagcgaagcggggtc

**L K R Q W Y T G K R Q W Y T G K R S G V** 170

601 gagcaagccgacgacggagacttggagcaacaatacaacaaacggcaatggtataccggt

**E Q A D D G D L E Q Q Y N K R Q W Y T G** 190

661 aaaagggccgacgaccttgctgatgctgctgacctagaaaagcggcagtggtacaccggg

**K R A D D L A D A A D L E K R Q W Y T G**  210

721 aaaaggcagtggtacaccggtaaacgcagcgatagtgaagacgagaaaaggcagtggtac

**K R Q W Y T G K R S D S E D E K R Q W Y** 230

781 accggtaaacgcagcgatagtgaagacgagaaaaggcagtggtacaccggtaaaagacaa

**T G K R S D S E D E K R Q W Y T G K R Q** 250

841 tggtataccggtagacgttaatcaaccaccaccactccttaatgaactgtcaaaatattt

**W Y T G R R *** 256

901 c**ctggatattgcggggctatta**

**Supplementary Figure 5. *A. rubens* thyrotropin-releasing hormone (TRH)-like precursor cDNA sequence.** The nucleotide sequence (lowercase, 922 bases) encoding the precursor protein (uppercase, 256 amino acid residues) is shown. Primers used for cloning are represented in bold and underlined text. The predicted signal peptide is represented in blue, fourteen putative TRH-type peptides are represented in red, C-terminal glycine (G) residues that are putative substrates for amidation are represented in orange and putative dibasic cleavage sites are represented in green. The asterisk shows the position of the stop codon. This cDNA sequence shares partial identity with a longer assembled transcript sequence, which has been deposited in the GenBank database under accession number KT601714. Nucleotides and amino acid residues that differ to the assembled transcript sequence are represented in underlined italics; these include a 90 nucleotide sequence encoding a 30 residue sequence in the C-terminal region of the precursor (between residues 219 and 249), which is not present in the assembled transcript sequence (probably due to an assembly error caused by the presence of repetitive DNA sequences in this transcript).

1 **ag**

3 **agtcactggagttaaga**agcccaagtcaccttataaggtaattttgtacagatggccgat

**M A D** 3

63 atgaggatgttaacactcactagcgtattagtctctctactcttcatggcagaaattcaa

**M R M L T L T S V L V S L L F M A E I Q** 23

123 agatgccaagggcagatacattacaagaatcctggatggggacctggtggtaaaaggagt

**R C Q G Q I H Y K N P G W G P G G K R S** 43

183 tcacacatgactggtagcaatgtattaaggaaacggcattggcgcgtggaatctgatcag

**S H M T G S N V L R K R H W R V E S D Q** 63

243 atgggtacagacagcatgcagaaagaacgaaacttgatcatgcttcaagaaattgcaaaa

**M G T D S M Q K E R N L I M L Q E I A K** 83

303 tctttggcaaagcaactggtagtaccaacgagtgaggacgacacagtcctggaccaatta

**S L A K Q L V V P T S E D D T V L D Q L** 103

363 acggtcgaccaatggcggcaggaagcagacgagataaatgacaacggttggaattaagcg

**T V D Q W R Q E A D E I N D N G W N *** 121

423 ggaaaagctctgaaatttgacaacaattatttagaatcaggaagaact**gaacaacttgat**

483 **acaggttc**

**Supplementary Figure 6. *A. rubens* gonadotropin-releasing hormone (GnRH)-type precursor cDNA sequence.** The nucleotide sequence (lowercase, 490 bases) encoding the precursor protein (uppercase, 121 amino acid residues) is shown. Primers used for cloning are represented in bold and underlined text. The predicted signal peptide is represented in blue, a putative GnRH-type peptide is represented in red, a C-terminal glycine (G) residue that is a putative substrate for amidation is represented in orange and a putative dibasic cleavage site is represented in green. The asterisk shows the position of the stop codon. This cDNA sequence is identical to part of a longer assembled transcript sequence, which has been deposited in the GenBank database under accession number KT601712.

1 **c**

2 **aaaggcaagggaagagatct**atataatacaatcatgaagcctacaacagttttgacgcta

**M K P T T V L T L** 9

62 gccgtcttctgcaccctttacaccatcatcacagccgcctcgatatccagggatgatgac

**A V F C T L Y T I I T A A S I S R D D D** 29

122 atgtttgacgtgacgggagacgatctgaggcaattagcaaagaaagtagacacatacgca

**M F D V T G D D L R Q L A K K V D T Y A** 49

182 agaaacaacgaaatacagtcactactaaaaagaaatggggaatctcgtggctgttcagga

**R N N E I Q S L L K R N G E S R G C S G**  69

242 ttcggcggctgtggggttctgactattggtcacaacgccgccatgcgcatgctcgctgaa

**F G G C G V L T I G H N A A M R M L A E** 89

302 tccaactcgcccttcggtgccagtggtccaggcaagagaaggagatcggttgacgctgta

**S N S P F G A S G P G K R R R S V D A V** 109

362 gcaaaccaagaggcgta**gaaaggattgaacaacgggag**

**A N Q E A *** 114

**Supplementary Figure 7. *A. rubens* calcitonin-type precursor (ArCTP) cDNA sequence.** The nucleotide sequence (lowercase, 399 bases) encoding the precursor protein (uppercase, 114 amino acid residues) is shown. Primers used for cloning are represented in bold and underlined text. The predicted signal peptide is represented in blue, a putative calcitonin-type peptide (with cysteine (C) residues underlined) is represented in red, a C-terminal glycine (G) residue that is a putative substrate for amidation is represented in orange and putative dibasic cleavage sites are represented in green. The asterisk shows the position of the stop codon. This cDNA sequence is identical to part of a longer assembled transcript sequence, which has been deposited in the GenBank database under accession number KT601715.

1 **g**

2 **tgtgcccggcatcagtt**tggcttaggagacatacactctgacaacacgtgagatatcatt

62 ttttatggttttactttcttcggagcatcgggtcagaatattctttgtataggggcaagg

122 cgaattgggtttttaaaccaatcggagagttttgcatattttgttcaggacgacattaca

182 ttcaccacaagggcctttacggtaggaaaacaacagaaactaaaggttgacgcagtcacc

242 atgaacgacctacagcggcttattctgttagtgtcccttggaacgttcgcccttctcctg

**M N D L Q R L I L L V S L G T F A L L L** 20

302 tgcctccccgcctgcaccgaggcgcaacctctaggcctgtttaaatttgaatacgacgat

**C L P A C T E A Q P L G L F K F E Y D D** 40

362 ttgttggaccccagttttgaagcggacgatcccaggaatccaaggagactttcgagacag

**L L D P S F E A D D P R N P R R L S R Q** 60

422 caaatcttaaggagactgaatgatttggcaatgtcacgctcaggatcaggaccaggttac

**Q I L R R L N D L A M S R S G S G P G Y** 80

482 acgattccaagaaaaaggcaaggcctgtctgtttcgcccatattcccaattcagaggatc

**T I P R K R Q G L S V S P I F P I Q R I** 100

542 cgtttgaacgcaatcgaacgggaccgtcaagaccaagtcgatcaggccgaggccaaccag

**R L N A I E R D R Q D Q V D Q A E A N Q** 120

602 ggcctttttcaaatcgccggacgcaagagatagggcaaatcataaaactaatgaccaaaa

**G L F Q I A G R K R *** 130

662 taggtgtttgcaatttgtttacagatttcgtcaggatattacacaggctgaattttaaaa

722 agggtaacaacggactttttgttatacacaactcggtttgcatctttgtgttcagacgcc

782 ccccataataggaccccaggcgaagcaggctttaaacagatgtgtgagttcact**caccac**

842 **tccacacggcag**

**Supplementary Figure 8. *A. rubens* corticotropin-releasing hormone (CRH)-like precursor cDNA sequence.** The nucleotide sequence (lowercase, 853 bases) encoding the precursor protein (uppercase, 130 amino acid residues) is shown. Primers used for cloning are represented in bold and underlined text. The predicted signal peptide is represented in blue, a putative CRH-type peptide is represented in red, a C-terminal glycine (G) residue that is a putative substrate for amidation is represented in orange and a putative dibasic cleavage sites are represented in green. The asterisk shows the position of the stop codon. This cDNA sequence is identical to part of a longer assembled transcript sequence, which has been deposited in the GenBank database under accession number KT601710.
